# The Impact of Priority Settings at the Start of COVID-19 Mass Vaccination on Subsequent Vaccine Uptake in Japan: One-Year Prospective Cohort Study

**DOI:** 10.2196/42143

**Published:** 2023-07-10

**Authors:** Daisuke Hori, Tsukasa Takahashi, Akihiko Ozaki, Takahiro Tabuchi

**Affiliations:** 1 Institute of Medicine University of Tsukuba Tsukuba Japan; 2 Department of Breast and Thyroid Surgery Jyoban Hospital of Tokiwa Foundation Iwaki Japan; 3 Cancer Control Center Osaka International Cancer Institute Osaka Japan

**Keywords:** cohort studies, SARS-CoV-2, COVID-19, Japan, vaccination, vaccination hesitancy, vaccines

## Abstract

**Background:**

Distributing COVID-19 vaccines to the public was an important task for the governments of each country. Because of various limitations, priority settings for vaccination were determined at the time of mass vaccination. However, trends between vaccine intention and uptake, as well as reasons for getting vaccinated or not getting vaccinated, among these groups were understudied, undermining verification of the legitimacy of priority selection.

**Objective:**

This study aims to illustrate a trend from prior COVID-19 vaccine intention, when the vaccine was not available, to the actual uptake within 1 year when all residents had access to the vaccine, to illustrate a change of reason for getting vaccinated or not getting vaccinated and to examine whether priority settings predicted subsequent vaccination uptake.

**Methods:**

Prospective cohort, web-based, self-administered surveys were conducted in Japan at 3 time points: February 2021, September to October 2021, and February 2022. In total, 13,555 participants (age: mean 53.1, SD 15.9 years) provided valid responses, with a 52.1% follow-up rate. On the basis of the information obtained in February 2021, we identified 3 types of priority groups: health care workers (n=831), people aged ≥65 years (n=4048), and those aged 18 to 64 years with underlying medical conditions (n=1659). The remaining patients were treated as nonpriority (n=7017). Modified Poisson regression analysis with a robust error estimated the risk ratio for COVID-19 vaccine uptake after adjusting for socioeconomic background, health-seeking behavior, attitude toward vaccines, and COVID-19 infection history.

**Results:**

In February 2021, a total of 5182 out of 13,555 (38.23%) respondents expressed their intention to get vaccinated. In February 2022, a total of 1570 out of 13,555 (11.6%) respondents completed the third dose and 10,589 (78.1%) respondents completed the second dose. Prior vaccine intention and subsequent vaccine coverage rates were higher in the priority groups. Protection of themselves and their families from potential infection was the most frequent reason for getting vaccinated, whereas concern about side effects was the most frequent reason for hesitation across the groups. Risk ratios for received, reserved, or intended for vaccination in February 2022 were 1.05 (95% CI 1.03-1.07) for the health care worker group, 1.02 (95% CI 1.005-1.03) for the older adult group, and 1.01 (95% CI 0.999-1.03) for the preexisting conditions group compared with the nonpriority group. Prior vaccine intention and confidence in vaccines were strong predictors of vaccine uptake.

**Conclusions:**

The priority settings at the start of the COVID-19 vaccination program had a significant impact on vaccine coverage after 1 year. The priority group for vaccination achieved higher vaccination coverage in February 2022. There was room for improvement among the nonpriority group. The findings of this study are essential for policy makers in Japan and other countries to develop effective vaccination strategies for future pandemics.

## Introduction

### Background

Since 2020, the wave of COVID-19 infection caused by SARS-COV-2 has affected populations intermittently around the world. To address this global challenge, COVID-19 vaccines were quickly developed, tested, licensed, and delivered worldwide in a relatively short time frame [1]. Vaccines are the most effective way to reduce the risk of COVID-19 infection, to bring herd immunity, and eventually to end the pandemic [2-4]. Being vaccinated contributes not only to preventing infection but also to reducing the severity, hospitalization, and mortality rates of COVID-19 [5-8]. In addition to the direct benefits to vaccine recipients, there are indirect benefits to those who are protected from the spread of COVID-19 infection. At the initial stage of vaccination, due to limitations in supply, logistics, and workforce, it was impossible to provide an equal opportunity for vaccination to all citizens at the same time. Prioritizing health care workers (HCWs) and people with medical vulnerabilities associated with poorer COVID-19 outcomes, such as older adults or those with chronic diseases, was common practice across countries [9-13]. Certainly, it was necessary to prioritize these populations when new vaccines were introduced to maintain the health care system and social function. However, at the same time, this practice might create artificial health disparities between priority and nonpriority groups. Attention should be paid to the manner in which the setting of priorities has affected the subsequent vaccine uptake situation.

The following paragraph describes an overview of the COVID-19 vaccine situation in Japan. Regarding countermeasures against COVID-19, there were no strict restrictions that involved penalties, such as those imposed during the lockdown in the United Kingdom. Instead, the government declared a state of emergency and called for citizens to refrain from actions that could spread the infection. It should also be noted that Japan has a universal health care system. COVID-19 vaccination was free of charge and was not mandatory. On February 14, the Pfizer vaccine was approved in Japan. The first dose of vaccination began on February 17, 2021, targeting HCWs in the National Hospital Organization, particularly those on the frontlines of COVID-19 patient care. Nationwide vaccination for HCWs began in March 2021. Mass vaccination for the following groups started in April: those aged ≥65 years at the end of March, those aged 18 to 64 years with preexisting conditions, and those who were working in nursing homes [14]. The government approved the Takeda or Moderna vaccine in May and the AstraZeneca vaccine in August. Although Japan started mass vaccination several months later than Western countries, vaccination coverage grew steadily once it began [15,16]. The third dose of vaccination for HCWs started in December 2021. As of April 2022, Japan has depended on imported vaccines supplied by Pfizer, Takeda or Moderna, and AstraZeneca that require 2-dose shots. By April 2022, more than half of the total population had received a booster dose, although vaccination rates among those in their 20s and 30s were reported to be low [17]. Those aged ≥60 years and those with underlying conditions were given the opportunity for a fourth vaccination dose, starting in May 2022, before HCWs.

Researchers have investigated the sociodemographic, psychological, behavioral, and social factors associated with vaccine intention or uptake status. In Japan, the following factors were related to vaccine intention or uptake: age [18-25], sex [18,20-25], employment status [18,22], marital status [18,19], income [18-20,22,23], educational attainment [18,19,22], underlying medical conditions [18-21,25], influenza vaccine history [19,24-26], medical checkup history [26], and COVID-19–related fear or anxiety [18-20,25,26]. Regarding attitudes toward the COVID-19 vaccine, concerns about the side effects of vaccines [25,26], confidence in vaccine effectiveness and public authorities [18,20,25], and pseudoscience [26] were linked to vaccine intention. Concern about adverse reactions is one of the most common reasons for vaccine hesitancy [21,22,25]. Although most of the existing literature relies on cross-sectional data, longitudinal studies on vaccine intention and subsequent uptake status are emerging worldwide [27-32]. These longitudinal studies demonstrate that the shift from initial intention to COVID-19 vaccination to actual uptake was influenced by the information about vaccine safety and efficacy that became available to the public. However, to date, no longitudinal studies have simultaneously observed the shift between those who are eligible for priority vaccination and those who are not. This is mainly because of the difficulty in recruiting a sufficient number of respondents in cohort studies.

### Objectives

Two series of web-based, prospective cohort studies are ongoing: the Japan Society and New Tobacco Internet Survey (JASTIS) and the Japan COVID-19 and Society Internet Survey (JACSIS). We derived 1-year longitudinal data from 3 time points of these surveys to address the following objectives: (1) to illustrate a trend from prior COVID-19 vaccine intention, when the vaccine was not available, to the actual uptake within 1 year when all residents had access to the vaccine; (2) to illustrate a change of reason for being vaccinated or not being vaccinated; and (3) to examine whether priority settings predicted vaccination uptake. The results are presented focusing on 3 types of vaccination priority and nonpriority groups at the rollout of mass vaccination. This approach allowed us to identify important trends over time and to examine whether the setting of priority groups was reasonable. Our study will also provide profound information for governments, health care policy makers, and health care providers to determine the target population for public health messages intended to promote vaccine uptake and how to tailor these messages for their specific audience.

## Methods

### Study Setting and Design

The data were derived from JASTIS from 2015 onward and JACSIS from 2020 onward. The study profiles have been described elsewhere [18,33]. Briefly, they comprised a series of web-based, self-administered cohort surveys that shared the same survey panel. The survey panel was drawn from approximately 2.2 million panelists at a Japanese internet research company (Rakuten Insight, Inc), which comprises individuals from diverse socioeconomic backgrounds (eg, marital status, educational level, and household income) to be a nationally representative sample of Japan. In JASTIS and JACSIS, sampling was stratiﬁed by sex, age, and prefecture of residence to be as nationally representative as possible. We used 3 time points to analyze the longitudinal data from the surveys conducted from February 8 to 25, 2021 (T1); September 27 to October 29, 2021 (T2); and February 1 to 28, 2022 (T3). All the respondents received a nominal incentive for survey completion at each time point.

### Study Participants

At T1, an email containing a link to the survey website was distributed to 32,827 candidates who participated in the same survey series conducted between 2015 and 2020. In total, 73.29% (24,059/32,827) of the participants answered the questionnaire. The survey continued to gather new panelists from the same internet research company until the number of respondents reached the targeted sample size (n=26,000). The respondents who answered that they were HCWs were classified as HCW group. The remaining respondents were classified into 3 groups: those who were aged ≥65 years, those who were aged 18 to 64 years with at least one preexisting condition, and those who were aged 18 to 64 years with no preexisting conditions. Preexisting conditions were defined as the following 14 conditions: hospital attendance for hypertension, diabetes mellitus, asthma, pneumonia or bronchitis, angina pectoris, myocardial infarction, stroke (including cerebral infarction and cerebral hemorrhage), chronic obstructive pulmonary disease, chronic kidney disease, chronic liver disease (excluding fatty liver and hepatitis), immune disorders and other diseases that cause immune deficiency (including steroid use), or cancer (including malignant tumor); sleep apnea identified by a medical checkup or physician; and a BMI ≥30 kg/m^2^. The exclusion criteria are summarized in Multimedia Appendix 1.

### Ethics Approval, Informed Consent, and Participation

Ethics approval for this study was obtained from the ethics committee of the Osaka International Cancer Institute (#20084-6) and the ethics committee of the University of Tsukuba, Institute of Medicine (#1737). All procedures were performed in accordance with the ethical standards of the institutional or national research committees and the 1964 Declaration of Helsinki and its later amendments, or comparable ethical standards. All participants provided web-based informed consent at registration. The study data were deidentified. For the research incentives, the participants earned points that could be used for shopping but the amount was not disclosed.

### Measures

#### Assessment of COVID-19 Vaccine Intention, Uptake, and Reasons for a Decision

In each survey, COVID-19 vaccine intention and uptake were assessed using a single-item question. The details are described in Multimedia Appendix 2. According to the response at T3, the participants were dichotomized into either a hesitant group or a received, reserved, or intended group. The hesitant group included participants who expressed that they did not want to get vaccinated or they would like to get vaccinated after waiting to see how it goes. The received, reserved, or intended group included participants who answered that they wanted to get vaccinated; had reserved a vaccination; or had received the first, second, and third (booster) dose of vaccine.

Each survey asked the received, reserved, or intended group for their reasons for getting vaccinated against COVID-19. The following sentence was only asked at T1: “It was recommended by SNS or the media.” The following sentences were only asked at T2 and T3: “It was recommended by the media,” “It was recommended by SNS,” and “It was recommended by a health care worker.” Each survey also asked the hesitant group about reasons for not getting vaccinated. The following sentences were only asked at T2 and T3: “Long-term side effects are unknown,” “I’m worried about short term side effects,” “I’m worried about death by the vaccines,” and “I don’t trust the vaccines’ approval process.” Multiple answers were allowed; therefore, the sum of the proportions for each reason did not necessarily add up to 100%.

#### Sociodemographic and Behavioral Factors and Vaccine-Related Scales

The following variables were derived from the T1 survey: age group, preexisting conditions, employment status, sex, marital status, educational background, occupation, annual household income, and influenza vaccination in the 2019/2020 season. The following variables were derived from the T2 survey: regular medical checkups before the COVID-19 pandemic, the vaccine hesitancy scale, the vaccine conspiracy beliefs scale, and the fear of COVID-19 scale. COVID-19 infection history and vaccine supplier details were derived from the T3 survey.

The vaccine hesitancy scale modified version [34,35] was used, which comprises 9 items, such as “Vaccines are important for my health,” scored on a 5-point Likert scale ranging from 1 (strongly disagree) to 5 (strongly agree). One item, “I do not need vaccines for diseases that are not that common anymore,” was omitted because COVID-19 was not convergent. The average scores for 2 subscales were calculated: 2 items for aversion to the risk of vaccination and 7 items for lack of confidence in the vaccine [36]. A higher average score, each ranging from 1 to 5, indicates a higher level of lack of confidence or risks. The Cronbach α was .62 for aversion to risk and .93 for lack of confidence.

The vaccine conspiracy beliefs scale was used, which comprises 7 items, such as “Vaccine safety data is often fabricated,” scored on a 7-point Likert scale from 1 (strongly disagree) to 7 (strongly agree) [35]. A higher average score, ranging from 1 to 7, indicates a higher level of conspiracy beliefs against the vaccine. The Cronbach α was .95.

The fear of COVID-19 scale was used, which comprises 7 items, such as “I am very afraid of coronavirus-19,” scored on a 5-point Likert scale from 1 (strongly disagree) to 5 (strongly agree) [37,38]. A higher average score, ranging from 1 to 5, indicates a higher level of fear of COVID-19. The Cronbach α was .84.

The score for the aversion to the risk of vaccination was classified into 2 groups by the median: “low” defined as a score lower than the median and “high” defined as a score equal to or higher than the median. Scores for the lack of confidence in the vaccine, conspiracy beliefs about the vaccine, and fear of COVID-19 were categorized into 3 groups by the tertile scores: “low” defined as a score below the lower tertile, “middle” defined as a score between the lower and higher tertiles, and “high” defined as a score higher than the higher tertile.

The participants were asked if they had been diagnosed with COVID-19 infection. Those who answered “Yes, within the last year” or “Yes, over a year ago” were defined as having a COVID-19 infection history. Participants were also asked if they had been hospitalized due to COVID-19.

Those who had received the vaccination at least once were asked which company supplied the vaccines they received. The possible answers were Pfizer, Takeda or Moderna, AstraZeneca, Johnson & Johnson, others, and “I don’t know.”

### Statistical Analysis

To identify factors associated with COVID-19 vaccine uptake at T3, we calculated the risk ratios (RRs) and 95% CIs using a modified Poisson regression analysis with a robust error variance [39]. The objective variable was vaccine uptake; those who had received vaccination, reserved vaccination, or intended to receive vaccination were coded as 1, and the hesitant individuals were coded as 0. In model 1, unadjusted RRs were calculated for the priority setting. In model 2, the following variables assessed at T1 were used for adjustment: prior COVID-19 vaccination intention, age group, employment status, number of preexisting conditions, sex, marital status, educational background, annual household income, and influenza vaccination in the 2019/2020 season. Model 3 was additionally adjusted for the following variables assessed at T2: regular medical checkups before the pandemic, aversion to the risk of vaccination, lack of confidence in the vaccine, conspiracy beliefs about the vaccine, and fear of COVID-19. Model 4 was additionally adjusted for COVID-19 infection history at T3. A sensitivity analysis was conducted, including age group, employment status, and number of preexisting conditions instead of vaccination priority settings.

Before conducting the modified Poisson regression analysis, the multiple regression analysis confirmed that there was no multicollinearity among the explanatory variables. The 2-sided significance level was set at 5%, and SPSS Statistics (version 28; IBM Corp) was used for all the analyses.

## Results

### Eligible Participants for Analysis

The flowchart of selecting eligible participants for analysis is illustrated in Multimedia Appendix 3. After excluding participants who matched the exclusion criteria or were lost to follow-up at each time point, a total of 13,555 individuals (age: mean 53.1, SD 15.9 years; 6519/13,555, 48.09% female) remained for analysis. They were older and had more male individuals than the excluded 12,445 individuals (age: mean 46, SD 16.9 years; 6231/12,445, 50.1% female). The overall valid follow-up rate was 52.1%. A total of 6538 individuals were eligible for each of the 3 types of vaccination priority: 1659 (25.37%) aged <65 years with underlying medical conditions (age: mean 51.5, SD 10.2 years; 540/1659, 32.55% female), 4048 (61.91%) aged ≥65 years (age: mean 71.3, SD 4.1 years; 1965/4048, 48.54% female), and 831 (12.71%) HCWs (age: mean 44.7, SD 12.6 years; 379/831, 45.6% female). A total of 7017 individuals did not meet any of the requirements for vaccination priority (age: mean 44, SD 12.4 years; 3635/7017, 51.80% female).

Table 1 shows the characteristics of the participants according to the priority settings at T1. At T3, the proportion of the hesitant group was highest in the nonpriority group (13.3%) and lowest in the priority group (4.5%) of older adults. Table S1 in Multimedia Appendix 4 summarizes the breakdown of underlying medical conditions among the priority group of those with preexisting conditions. Hypertension (n=779) and sleep apnea (n=409) were the most common preexisting conditions. The proportion of the received, reserved, or intended group ranged from 86.5% among patients with angina pectoris to 98.7% among patients with immune deficiency. Table S2 in Multimedia Appendix 4 shows the breakdown of jobs among the priority group of HCWs. There were 110 nurses, 63 pharmacists, and 55 physicians. Midwives (n=7) exhibited relatively lower vaccine coverage of 71.4%. Tables S1 to S5 in Multimedia Appendix 5 summarize the COVID-19 vaccine coverage rate stratified by COVID-19 infection history or hospitalization over 1 year. Higher RRs indicated that those who had been infected with COVID-19 in the past were more likely to receive vaccines than those who had not been infected. Across all 4 groups, the RRs ranged from 0.90 to 1.10.

Table 2 demonstrates the kind of COVID-19 vaccines that were inoculated to the respondents by T3. Most people had been vaccinated with Pfizer vaccines.

**Table 1:** Characteristics of the respondents stratified by the COVID-19 vaccination priority settings at T1 (February 2021; the percentages in parentheses in each category vertically add up to 100).

Characteristics		Total (N=13,555), n (%)	Nonpriority (n=7017), n (%)	Aged 18-64 years with preexisting conditions, non-HCW^a^ (n=1659), n (%)	Aged ≥65 years, non-HCW (n=4048), n (%)	HCW (n=831), n (%)
**Prior COVID-19 vaccination intention^b^**						
	Intended	5182 (38.23)	1966 (28.02)	683 (41.17)	2130 (52.62)	403 (48.5)
	Wait and see	6936 (51.17)	4133 (58.9)	829 (49.97)	1644 (40.61)	330 (39.71)
	Refused	1437 (10.6)	918 (13.08)	147 (8.86)	274 (6.77)	98 (11.79)
**Age group (years)^b^**						
	18-34	2035 (15.01)	1713 (24.41)	119 (7.17)	0 (0)	203 (24.42)
	35-44	2052 (15.14)	1601 (22.82)	234 (14.1)	0 (0)	217 (26.11)
	45-54	2774 (20.24)	2029 (28.91)	529 (31.89)	0 (0)	216 (25.99)
	55-64	2600 (19.18)	1674 (23.86)	777 (46.83)	0 (0)	149 (17.93)
	65-80	4094 (30.2)	0 (0)	0 (0)	4048 (100)	46 (5.53)
**Employment status^b^**						
	Employed, non-HCW	7209 (53.18)	4997 (71.21)	1221 (73.6)	991 (24.48)	0 (0)
	Employed, HCW	831 (6.13)	0 (0)	0 (0)	0 (0)	831 (100)
	Unemployed	2034 (15)	488 (6.95)	187 (11.27)	1359 (33.57)	0 (0)
	Not working (student, homemaker, or retiree)	3481 (25.68)	1532 (21.83)	251 (15.13)	1698 (41.95)	0 (0)
**Number of preexisting conditions^b^**						
	0	9860 (72.74)	7017 (100)	0 (0)	2177 (53.78)	666 (80.14)
	>1	3695 (27.26)	0 (0)	1659 (100)	1871 (46.22)	165 (19.86)
**Sex^b^**						
	Male	7036 (51.91)	3382 (48.2)	1119 (67.45)	2083 (51.46)	452 (54.39)
	Female	6519 (48.09)	3635 (51.8)	540 (32.55)	1965 (48.54)	379 (45.61)
**Marital status^b^**						
	Single, divorced, or widowed	4945 (36.48)	3133 (44.65)	606 (36.53)	886 (21.89)	320 (38.51)
	Married	8610 (63.52)	3884 (55.35)	1053 (63.47)	3162 (78.11)	511 (61.49)
**Educational background^b^**						
	Others	6811 (50.25)	3324 (47.37)	829 (49.97)	2290 (56.57)	368 (44.28)
	4-year college, university, or graduate	6744 (49.75)	3693 (52.63)	830 (50.03)	1758 (43.43)	463 (55.72)
**Household income (million; yen)^b^**						
	<5	5530 (40.8)	2377 (33.87)	576 (34.72)	2347 (57.98)	230 (27.68)
	5-10	4048 (29.86)	2369 (33.76)	571 (34.42)	779 (19.24)	329 (39.59)
	≥10	1383 (10.2)	832 (11.86)	226 (13.62)	186 (4.59)	139 (16.73)
	I do not know or prefer not to answer.	2594 (19.14)	1439 (20.51)	286 (17.24)	736 (18.18)	133 (16)
**Influenza vaccination in the 2019/2020 season^b^**						
	No	8246 (60.83)	4762 (67.86)	1018 (61.36)	2130 (52.62)	336 (40.43)
	Yes	5309 (39.17)	2255 (32.14)	641 (38.64)	1918 (47.38)	495 (59.57)
**Regular medical checkups before the pandemic^c^**						
	No	4612 (34.02)	2711 (38.63)	487 (29.36)	1157 (28.58)	257 (30.93)
	Yes	8943 (65.97)	4306 (61.36)	1172 (70.64)	2891 (71.42)	574 (69.07)
**Aversion to the risk of vaccination^c^**						
	Low, 1.00-3.00	6587 (48.59)	2984 (42.52)	801 (48.28)	2370 (58.55)	432 (51.99)
	High, 3.01-5.00	6968 (51.4)	4033 (57.47)	858 (51.72)	1678 (41.45)	399 (48.01)
**Lack of confidence in the vaccine^c^**						
	Low, 1.00-1.99	4080 (30.1)	1660 (23.66)	507 (30.56)	1666 (41.16)	247 (29.72)
	Middle, 2.00-2.43	5458 (40.26)	2791 (39.77)	644 (38.82)	1735 (42.86)	288 (34.66)
	High, 2.44-5.00	4017 (29.63)	2566 (36.57)	508 (30.62)	647 (15.98)	296 (35.62)
**Conspiracy beliefs about the vaccine^c^**						
	Low, 1.00-3.27	4253 (31.37)	1810 (25.79)	504 (30.38)	1673 (41.33)	266 (32.01)
	Middle, 3.28-4.00	6399 (47.21)	3566 (50.82)	784 (47.26)	1667 (41.18)	382 (45.97)
	High, 4.01-5.00	2903 (21.42)	1641 (23.39)	371 (22.36)	708 (17.49)	183 (22.02)
**Fear of COVID-19^c^**						
	Low, 1.00-2.27	3756 (27.71)	2135 (30.43)	398 (23.99)	973 (24.04)	250 (30.08)
	Middle, 2.28-3.00	6638 (48.97)	3350 (47.74)	824 (49.67)	2082 (51.43)	382 (45.97)
	High, 3.01-7.00	3161 (23.32)	1532 (21.83)	437 (26.34)	993 (24.53)	199 (23.95)
**COVID-19 infection history^d^**						
	None	13,264 (97.85)	6848 (97.59)	1607 (96.87)	4018 (99.26)	791 (95.19)
	Yes	291 (2.15)	169 (2.41)	52 (3.13)	30 (0.74)	40 (4.81)
**Vaccine uptake status^d^**						
	Third dose vaccinated	1570 (11.58)	225 (3.21)	71 (4.28)	954 (23.57)	320 (38.51)
	Second dose vaccinated	10,589 (78.12)	5804 (82.71)	1430 (86.2)	2906 (71.79)	449 (54.03)
	First dose vaccinated	57 (0.42)	41 (0.58)	7 (0.42)	5 (0.12)	4 (0.48)
	Reserved or intended	21 (0.15)	15 (0.21)	4 (0.24)	0 (0)	2 (0.24)
	Wait and saw	443 (3.27)	334 (4.76)	42 (2.53)	50 (1.23)	17 (2.05)
	Refused	875 (6.45)	598 (8.52)	105 (6.33)	133 (3.28)	39 (4.69)
**Dichotomized vaccine uptake status^d^**						
	Received, reserved, or intended	12,237 (90.28)	6085 (86.72)	1512 (91.14)	3865 (95.48)	775 (93.26)
	Hesitant	1318 (9.72)	932 (13.28)	147 (8.86)	183 (4.52)	56 (6.74)

^a^HCW: health care worker.

^b^The variables were derived from T1 (February 2021).

^c^The variables were derived from T2 (September to October 2021).

^d^The variables were derived from T3 (February 2022).

**Table 2:** Summary of the COVID-19 vaccine suppliers that the respondents received by T3 (February 2022)^a^.

COVID-19 vaccine supplier	Total (n=12,216), n (%)	Nonpriority (n=6070), n (%)	18-64 years with preexisting conditions, non-HCW^b^ (n=1508), n (%)	≥65 years, non-HCW (n=3865), n (%)	HCW (n=773), n (%)
Pfizer	9421 (77.12)	4146 (68.3)	1144 (75.86)	3482 (90.09)	649 (83.96)
Takeda or Moderna	2860 (23.41)	1834 (30.21)	359 (23.81)	545 (14.1)	122 (15.78)
AstraZeneca	47 (0.38)	24 (0.39)	7 (0.46)	8 (0.21)	8 (1.03)
Others	28 (0.23)	16 (0.26)	4 (0.26)	5 (0.13)	3 (0.38)
I don’t know	218 (1.78)	126 (2.07)	25 (1.66)	53 (1.37)	14 (1.81)

^a^Only those who had received at least 1 dose of the COVID-19 vaccine were asked to respond. Multiple answers were allowed.

^b^HCW: health care worker.

### Shift From Prior COVID-19 Vaccine Intention to Actual Vaccine Uptake 1 Year Later

**Figure 1:**
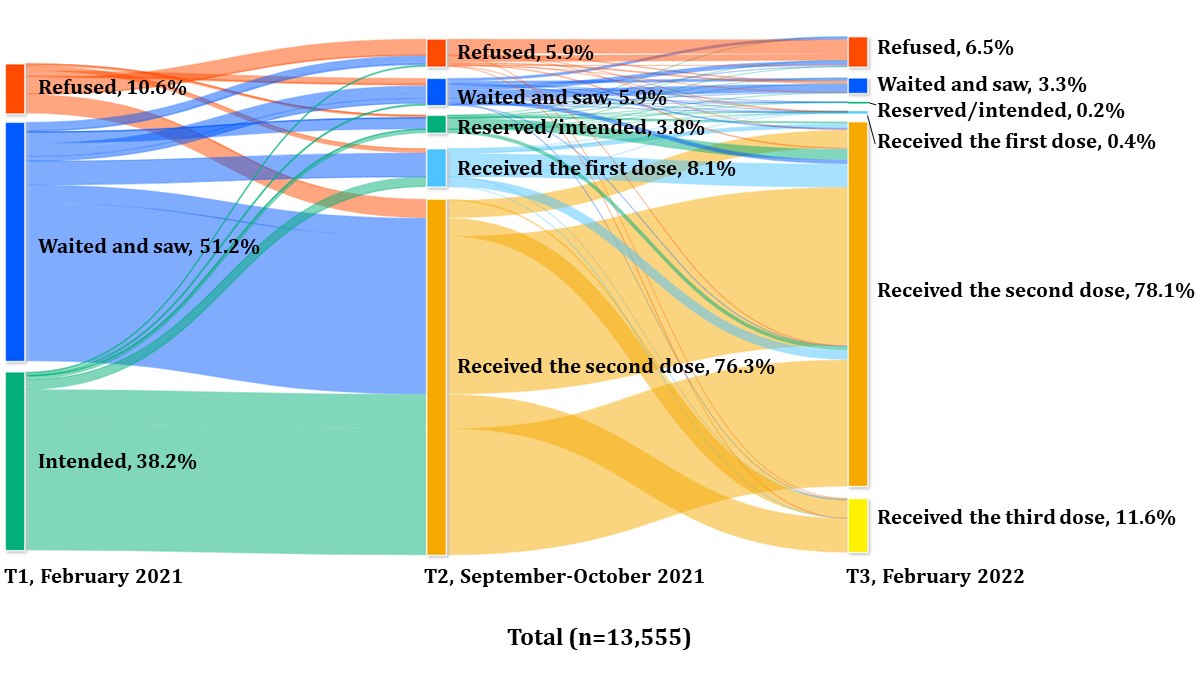
Shift from prior COVID-19 vaccine intention in February 2021 to actual vaccine uptake in February 2022, including the nonpriority group and the 3 priority groups. ChartExpo™ was used to illustrate the Sunkey diagram.

As illustrated in Figure 1, at T1, a total of 6936 out of 13,555 (51.17%) respondents answered, “I would like to get vaccinated after waiting to see how it goes.” At T2, a total of 10,339 out of 13,555 (76.27%) respondents had received 2 vaccination shots. At T3, a total of 10,589 out of 13,555 (78.12%) respondents completed 2 shots and 1570 (11.58%) respondents completed a third (booster) shot of vaccination. Figures S1 to S4 in Multimedia Appendix 6 showed the results stratified by the nonpriority group and the 3 priority groups. Prior intention to get vaccinated was lowest among the nonpriority group and highest in the older adult group. At T3, the percentage of those who had received the second dose/third dose of vaccine was 82.7%/3.2% in the nonpriority group, 86.2%/4.3% in the group with preexisting conditions, 71.8%/23.6% in the group aged ≥65 years, and 54%/38.5% in the HCW group.

### Shift in the Reasons for Receiving or Not Receiving COVID-19 Vaccination

**Figure 2:**
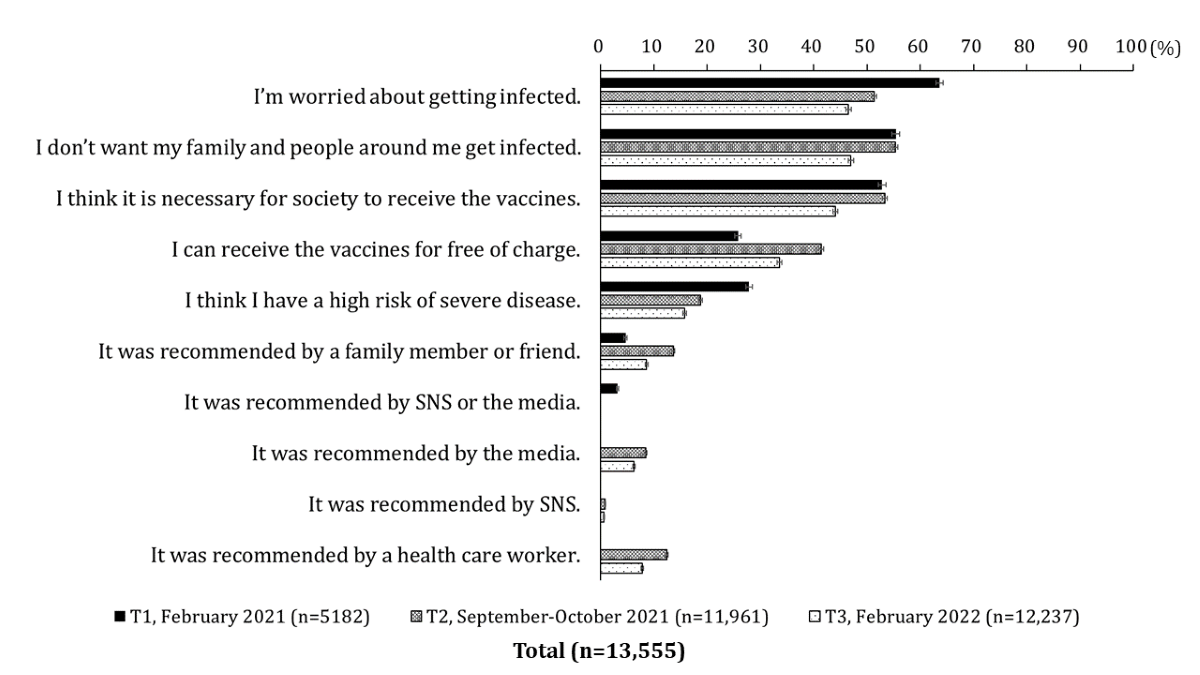
Shift in reason for getting vaccinated against COVID-19 from February 2021 to February 2022, including the nonpriority group and the 3 priority groups. Respondents were those who had intended or reserved to receive the vaccines or those who had received the vaccines at least once at each time point. The following sentences was asked only at T1: “It was recommended by SNS or the media.” The following sentences were only asked at T2 and T3: “It was recommended by the media,” “It was recommended by SNS,” and “It was recommended by a health care worker.”.

Figure 2 summarizes the shift in the reasons for getting vaccinated among those who received vaccination, who reserved vaccination, or who were intended for vaccination. The top reasons for getting vaccinated were worry about infecting themselves, their family, or others around them, followed by social norms. The percentage of those who expressed that they were worried about contracting COVID-19 gradually declined (63.6%, 51.4%, and 46.6%, respectively). Figures S1 to S4 in Multimedia Appendix 7 show the results for each priority group and the nonpriority group. There were intergroup characteristics in the reasons for getting vaccinated: the preexisting conditions and older adult groups were more likely to get vaccinated because they thought they had a high risk of contracting severe COVID-19 infection. The HCW group had the lowest number of reasons for getting vaccinated.

**Figure 3:**
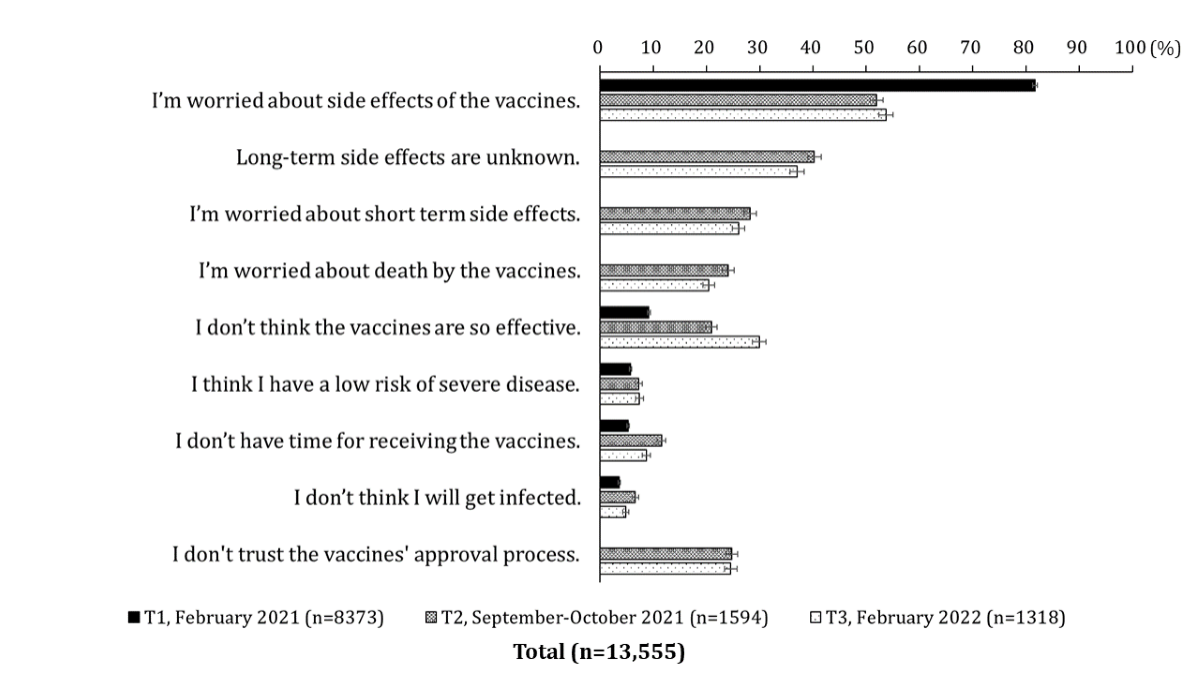
Shift in reason for not getting vaccinated against COVID-19 from February 2021 to February 2022, including the nonpriority group and the 3 priority groups. Respondents were those who chose to wait and see or refused to receive the vaccines at each time point.The following sentences was asked only at T2 and T3: “Long-term side effects are unknown,” “I’m worry about short term side effects,” “I’m worried about death by the vaccines,” and “I don’t trust the vaccines’ approval process.”.

The reasons for not getting vaccinated among the hesitant group are shown in Figure 3. The most common reason for not getting vaccinated among the unvaccinated people was concerns about side effects at all 3 time points. The percentages were highest when the vaccination program had not yet begun at T1, declined at T2, and remained at the same level at T3 (81.7%, 51.9%, and 53.7%, respectively). Concerns about long-term side effects were raised more frequently than those about short-term side effects. The percentage who argued that the COVID-19 vaccine was ineffective gradually increased over the course of the year (9.2%, 21%, and 29.9%, respectively). Figures S1 to S4 in Multimedia Appendix 8 show the results for the nonpriority group and each priority group. At T3, the percentage of respondents who answered “I’m worried about side effects of the vaccines” was highest in the priority group with underlying medical conditions. The proportion of those who said “I don’t trust the vaccines’ approval process” was highest in the priority group of adults aged ≥65 years.

### Priority Settings at the Start of Mass Vaccination and Subsequent COVID-19 Vaccine Uptake 1 Year Later

Table 3 summarizes the results of the Poisson regression analyses. In model 4, significantly higher RRs for getting vaccinated were observed in the priority group of adults aged ≥65 years (RR 1.02, 95% CI 1.005-1.03) and HCWs (RR 1.05, 95% CI 1.03-1.07).

A sensitivity analysis was performed by replacing the priority group with age group, employment status, and number of preexisting conditions. The results are shown in Multimedia Appendix 9. In model 4, prior willingness for COVID-19 vaccination, a lower level of aversion to the risk of vaccination, a higher level of confidence in the vaccine, and a lower level of conspiracy beliefs about the vaccine exhibited higher RRs, which indicated a higher likelihood of getting immunization. Those who had the following characteristics were more likely to receive COVID-19 vaccination: being older, female, higher educational level, higher household income, having received influenza vaccination in the 2019/2020 season, and regular medical checkups before the pandemic. In contrast, statistically significantly lower RRs were observed in those who were unemployed or not working and those who had a lower level of fear of COVID-19.

**Table 3:** Risk ratios (RRs) for COVID-19 vaccination uptake status at T3 (February 2022) by priority settings at T1 (February 2021).^a^

	Nonpriority, RR (95% CI)	18-64 years with preexisting conditions, non-HCW^b^, RR (95% CI)	≥65 years, non-HCW, RR (95% CI)	HCW, RR (95% CI)
Model 1	Reference	1.05 (1.03-1.07)	1.10 (1.09-1.11)	1.08 (1.05-1.10)
Model 2	Reference	1.03 (1.01-1.04)	1.05 (1.04-1.07)	1.04 (1.02-1.06)
Model 3	Reference	1.02 (0.999-1.03)	1.02 (1.004-1.03)	1.05 (1.03-1.07)
Model 4	Reference	1.01 (0.999-1.03)	1.02 (1.005-1.03)	1.05 (1.03-1.07)

^a^Modified Poisson regression analysis with a robust error variance was performed. A higher RR indicated a higher likelihood of receiving, reserving, or intending to vaccinate at T3. Model 1 was unadjusted. Model 2 was adjusted for prior vaccination intention, sex, marital status, educational background, annual household income, and influenza vaccination during the 2019/2020 season. In addition to model 2, model 3 was adjusted for regular medical checkups before the pandemic, aversion to the risk of vaccination, lack of confidence in the vaccine, conspiracy beliefs about the vaccine, and fear of COVID-19. In addition to model 3, model 4 was adjusted for COVID-19 infection history.

^b^HCW: health care worker.

## Discussion

### Principal Findings

Prompt distribution of COVID-19 vaccines to the public was one of the global goals to be accomplished in 2021. In this study conducted in Japan, we reported on the shift from COVID-19 vaccine intention to actual uptake and the reasons for getting or refusing vaccination in a 1-year period. In February 2021, a total of 5182 out of 13,555 (38.23%) participants expressed that they intended to take the vaccine, 6936 (51.17%) chose to wait and see, and 1437 (10.6%) refused to get vaccinated. One year later, >90% (12,216/13,555) of the participants had received at least 1 dose of the vaccine. There were considerable differences in uptake across the nonpriority group and the 3 typical vaccine priority groups. We confirmed that COVID-19 vaccination uptake rates were higher among priority groups than the nonpriority group in line with recent reports [17]. This trend continued for 1 year after the start of mass vaccination. Overall, protection against infection for themselves, their families, and the people around them was cited as the main reason for seeking vaccination. In contrast, concern about side effects was cited as the main reason for avoiding vaccination. We also elucidated the factors that predicted, or were associated with, vaccine uptake. As hypothesized, prioritized groups at the initial stage of the COVID-19 mass vaccination program exhibited a higher vaccine uptake rate after adjusting for sociodemographic background, health-seeking behavior, and attitude toward the vaccine. To our knowledge, this is the first longitudinal study to illustrate these results simultaneously by priority groups and nonpriority groups with the advantage of a large cohort sample.

The vaccination coverage rate in our sample was higher than that reported in the official Japanese vaccination statistics, which stated that approximately 75% of the total population had received at least 1 dose by the end of February 2022 [40]. Therefore, caution should be exercised when interpreting and generalizing these results. Although stratiﬁed sampling was used to get as close to a nationally representative population as possible, the participants were registered monitors of an internet research company, and sampling bias was inevitable. For example, it is difficult to reach those who have no access to the internet, such as the institutionalized, poor, or homeless. In addition, the follow-up rate was slightly higher than 50%. It is also possible that the participants at the baseline died of COVID-19 and dropped out. Our current sample may comprise more older and health-oriented people than the actual population. These factors could lead to the overestimation of vaccination coverage. Nevertheless, a web-based survey targeting registered monitors was a feasible and frequently used research method during the COVID-19 pandemic. It should also be noted that this study focused on priority settings at the start of vaccination in the spring of 2021. Governments around the world made changes to their priority conditions as the vaccines became more widely distributed; for instance, lowering the age threshold for the older adult group [11]. The Japanese government did not set a priority for the third (booster) vaccination, which started in December 2021.

Overall, almost all of those who intended or chose to wait and see to receive the vaccine before the vaccine rollout actually received the vaccine after it became available. A large intention-to-action gap was observed in the past in relation to vaccination against H1N1 influenza [41,42]; however, the gap appears to be much smaller for COVID-19 vaccines [27,29,31]. Approximately half of those who had initially refused the vaccine continued to refuse, whereas the other half changed their intentions and accepted vaccination. The persistent refusers across the 3 time points were found more in the nonpriority group and the priority group with preexisting conditions. It should be noted that, among the hesitant individuals in these 2 groups, the percentage of those who refused vaccination increased between T2 and T3. Whether they remain unvaccinated or change their mind afterward remains to be observed. The highest vaccination coverage rates were found among the priority groups of older adults and HCWs. More than 90% of participants in these groups completed 2-dose vaccination within half a year. This tendency was also confirmed by the multivariable analysis. The older adult group was considered one of the most susceptible groups to severe COVID-19 infection, and participants in this group were the most eager to get vaccinated, even before the vaccination program began. This finding is in line with previous studies that have reported that older age groups were more willing to get vaccinated [18-26].

Concerns about side effects in general remained the most common reason for vaccine hesitancy or resistance within the year. This result is in line with previous studies in Japan [21,22,25] and in other countries [43-46]. We demonstrated that this trend continued from before the start of mass vaccination to 1 year later. We further illustrated that the number of participants who thought the COVID-19 vaccine was ineffective increased gradually in the vaccine hesitant group. This may be because the COVID-19 pandemic has not yet settled despite increased vaccination rates. This may also be due to outbreaks of the omicron variants, which were reported to be highly infectious and resistant to currently available vaccines [47,48]. There were some intergroup differences among the groups. In the older adults and preexisting conditions groups, more respondents cited fear of severe COVID-19 and awareness of the need for vaccination as their reason for getting vaccinated. Despite having the second highest coverage rate, the HCW group cited the fewest reasons for vaccination. This group might have benefited from the availability of vaccines at their workplace, although the current survey did not provide information on where they were vaccinated. Future studies should consider the convenience of time and location for vaccination, which can affect vaccine hesitancy [49,50].

There were slight differences in subsequent vaccine uptake between those who had a history of COVID-19 infection and those who had no history at T1. The same was true for a history of COVID-19 infection at T3. It should be noted that during the surveyed period, from February 2021 to February 2022, it is unclear from the current data which came first, the infection or vaccination. There would have been both prevaccination and breakthrough infections during the surveyed period. The current results do not support the existing literature in other countries [31,51], which suggested that those who had previously been infected were less anxious about reinfection and had less interest in vaccination. However, vaccination after recovery from COVID-19 is reported to still be effective in preventing reinfection [52]. This inconsistency could be due to the small number of infected individuals in the current data set. Indeed, despite being a superaging society, Japan’s COVID-19 mortality rate is among the lowest in the world [53,54]. In our sample, the highest percentage of COVID-19 infection history was found in the HCW group, who were considered to have the most frequent contact with patients with COVID-19 and also the most frequent infection testing. This finding underlines the need to prioritize vaccination for HCWs [55]. In contrast, the older adults group had the lowest percentage of COVID-19 infection history. Together with high vaccination coverage, it is fair to say that the vaccination campaign targeting the older adult population was successful. The nonpriority group had the largest number of infected cases, although the percentage was smaller than that of the HCW and preexisting conditions groups.

Those who were younger, were unemployed, were unmarried, had lower educational attainment, had lower income, had a presence of underlying medical conditions, had not had an influenza vaccination, and had not attended regular health checkups before the start of mass vaccination exhibited a lower vaccine uptake rate 1 year later. The current results corroborate a number of cross-sectional studies in Japan that have reported an association between these factors with vaccine hesitancy [18-26]. Our findings were consistent with a number of studies in other countries that have elucidated factors related to vaccine intention and uptake [56-58]. We have demonstrated the importance of conducting vaccine promotion campaigns for these populations. Prior favorable vaccine intention and more positive attitudes about the safety and effectiveness of the vaccine significantly improved the likelihood of COVID-19 vaccine uptake. Those who believed that there was a conspiracy behind vaccines had a lower incidence of immunization. The low level of fear of COVID-19 was also associated with being vaccine hesitant. These results were congruent with previous studies [27,29,31,56,57,59]. In particular, prior intention for vaccination and confidence in vaccines were the strongest predictors of subsequent vaccine uptake. These findings support the idea that bridging the vaccination intention-to-action gap could be accomplished by overcoming concerns regarding vaccine safety [1,60].

### Clinical Implications

This study has important clinical implications. The public authorities need to start by acknowledging the disparities in vaccine uptake in the nonpriority group, including younger healthy adults. In Japan, the largest percentage of confirmed cases of COVID-19 was among individuals aged under 30 years [61], highlighting their significance as key players in COVID-19 transmission. Matrajt et al [62] claimed that switching the vaccine allocation target to the high-transmission groups from the high-risk groups would achieve high vaccine effectiveness, as is the case with influenza vaccines [63,64]. In other words, it is possible to reduce the number of patients with COVID-19 in the older adults group by controlling the number of patients in the young adults group. Therefore, it is essential to keep examining whether the vaccine priority settings were appropriate for maximizing vaccine effectiveness. In promoting vaccine uptake, the content of the message should be tailored to each population group, taking into account their attitudes toward vaccines. When targeting young and healthy groups, those responsible for delivering the message should recognize the concerns of these groups about vaccine safety and effectiveness. Changing the perception of vaccine risk would be a key strategy in promoting vaccination. Needless to say, the development of new vaccines that have fewer side effects and are more effective against new types of mutation is expected. Our results also suggest that it is important to begin the promotion of vaccine acceptance before the introduction of new vaccines. Even after COVID-19 has converged, other emerging infectious diseases could lead to pandemics in the future. One possible way to reduce vaccine hesitancy in introducing new vaccines is to increase the number of people who receive influenza vaccination or undergo medical checkups regularly. These behaviors would reduce the barriers to vaccination if a new virus emerged for which vaccines are required [65].

### Limitations

Despite the abovementioned strengths of this study, there are some limitations that should be noted. First, vaccine-related scales such as the vaccine hesitancy scale were only assessed at T2. At that time, some respondents had already received vaccination, while others had not. Therefore, we were unable to draw conclusions regarding the direction of causality between these variables. Second, due to the nature of self-administered surveys, recall bias and reporting bias may have occurred. Third, the classification of the priority groups was not completely accurate. For example, although the severity of each disease was set as a condition for priority, information on this was not obtained. As a proxy, a hospital visit for the disease was used to define the group. In addition, individuals who reached the age of 65 years in March 2021 were misclassified into the nonpriority group; however, the number of relevant participants was estimated to be small enough to affect the result. Participants working in a nursing home, one of the vaccination priority groups, could not be identified. Finally, we could not distinguish within-group differences in vaccine priority, such as frontline workers or office workers in a hospital.

### Conclusions

Our findings demonstrate that the priority settings at the start of the COVID-19 vaccination program had a significant impact on vaccine coverage 1 year later. Japan achieved a high rate of vaccine uptake, resulting in a low COVID-19 mortality rate at the start of 2022. However, things took a drastic turn in 2022 with the outbreak of the omicron variant, which was a complete change from the year 2021. At its peak in August 2022, there were >250,000 cases and >300 deaths per day. Japan experienced another surge in infections in January 2023. After its peak had passed, the government downgraded COVID-19 to category 5 of infectious disease, the same rank as seasonal influenza, in May 2023. Although the threat of COVID-19 appears to have subsided for the moment, there remains the possibility that new emerging infectious diseases will arise and cause unprecedented situations. Further research is necessary to determine the effectiveness of vaccine distribution procedures in preventing COVID-19 from 2022 to 2023. Such efforts are essential for reducing the impact of the next pandemic on individuals and society.

## Appendix

Multimedia Appendix 1 Exclusion criteria for analysis.

Multimedia Appendix 2 Summary of response options for COVID-19 vaccine intention or uptake for each time point, as well as classification of uptake status at T3 for the following modified Poisson regression analysis.

Multimedia Appendix 3 Flowchart of eligible participants for analysis.

Multimedia Appendix 4 Summary of preexisting conditions among the non–health care worker priority group aged 18-64 years with preexisting conditions, as well as the profession of those in the health care worker priority group.

Multimedia Appendix 5 COVID-19 infection over 1 year and risk ratios for COVID-19 vaccine uptake.

Multimedia Appendix 6 Shift from prior COVID-19 vaccine intention in February 2021 to actual vaccine uptake in February 2022, stratified by the nonpriority group and the 3 priority groups.

Multimedia Appendix 7 Shift in reason for getting vaccinated against COVID-19 from February 2021 to February 2022, stratified by the nonpriority group and the 3 priority groups.

Multimedia Appendix 8 Shift in reason for not getting vaccinated against COVID-19 from February 2021 to February 2022, stratified by the nonpriority group and the 3 priority groups.

Multimedia Appendix 9 Risk ratios for COVID-19 vaccination uptake status at T3 (February 2022) using sociodemographic factors, behavioral factors, and vaccine hesitancy–related scales.

## Abbreviations

HCW: health care worker
JACSIS: Japan COVID-19 and Society Internet Survey
JASTIS: Japan Society and New Tobacco Internet Survey
RR: risk ratio

## Data Availability

The data sets generated or analyzed during this study are available from the corresponding author upon reasonable request.
