# Supplementary material for: The Impact of Priority Settings at the Start of COVID-19 Mass Vaccination on Subsequent Vaccine Uptake in Japan: One-Year Prospective Cohort Study
Source: JMIR Public Health Surveill. 2023 Jul 10;9:e42143. doi: 10.2196/42143 (PMC10337369; doi:10.2196/42143)
Supplement: Multimedia Appendix 1 [file publichealth_v9i1e42143_app1.docx]

This is a Multimedia Appendix to a full manuscript published in the J Med Internet Res. For full copyright and citation information see http://dx.doi.org/10.2196/42143

**Exclusion criteria for analysis.**

Respondents who had provided invalid responses for three conditions were excluded in each timepoint: 1) those who did not follow the dummy instruction, “choose the second option from the bottom of a list”, 2) those who answered they sometimes or almost every day use all of the following nine substances: alcohol, sleeping pills/antianxiety drug, medical narcotics for cancer pain, medical narcotics for all pain except cancer, non-medical narcotics, organic solvents, dangerous drugs, marijuana, and stimulant/cocaine/heroin; or 3) those who answered they currently had all of the following nine comorbidities: hypertension, diabetes mellitus, asthma, atopic dermatitis, angina pectoris, myocardial infarction, stroke/cerebral infarction/cerebral hemorrhage, cancer/malignant tumor, and chronic pain.

At T1, February 2021, those who said they had already been vaccinated, those who were younger than 18 years old, those who had abnormal weight (less than 30 kg, or 150 kg and above) or abnormal height (less than 130 cm) were excluded. Among the health care worker group, those who worked less than 30 hours a week or those who were too young for their job (e.g., at 18 years old it is not possible to be a medical doctor) were excluded. Those who stated that they were not health care workers but answered “I would get vaccinated because I am health care worker” were also excluded.

At T2, September-October 2021, and T3, February 2022, those who said they would not get vaccinated because of an allergy/comorbidity and those who had received a single-shot type vaccine were excluded. At T3, we excluded those who had provided contradictory answers regarding vaccination (e.g. those who said they had not yet been vaccinated at T3, although they had reported receiving at least one vaccination at T2).
