# Supplementary material for: The Impact of Priority Settings at the Start of COVID-19 Mass Vaccination on Subsequent Vaccine Uptake in Japan: One-Year Prospective Cohort Study
Source: JMIR Public Health Surveill. 2023 Jul 10;9:e42143. doi: 10.2196/42143 (PMC10337369; doi:10.2196/42143)
Supplement: Multimedia Appendix 2 [file publichealth_v9i1e42143_app2.docx]

This is a Multimedia Appendix to a full manuscript published in the J Med Internet Res. For full copyright and citation information see http://dx.doi.org/10.2196/42143

**Table S1.** Summary of answering options to inquire about COVID-19 vaccine intention/uptake for each timepoint, and classification of uptake status at T3 (February 2022) for the following modified Poisson regression analysis. ^a^

| T1, February 2021 | T2, September-October 2021 | T3, February 2022 | Classification of vaccine uptake status at T3 |
| --- | --- | --- | --- |
| I don’t want to get vaccinated. | | | 0: Hesitant |
| I want to get vaccinated after waiting to see how it goes. | | |  |
| I want to get vaccinated. | I want to get vaccinated / I have reserved my first vaccination dose | | 1: Received, reserved, or intended |
| N/A | Although I would like to get vaccinated, I'm unable to do so due to allergy/comorbidity. | | Excluded from the analysis |
| N/A | I have had one dose of vaccine (single-shot type). | I have had one dose of vaccine (single-shot type). I don’t want to get the next one. |  |
| N/A |  | I have had one dose of vaccine (single-shot type). I want to get the next one. |  |
| N/A |  | I have already had one dose of vaccine (single-shot type) and a booster shot. |  |
| N/A | Although I have had my first dose of vaccine, I did not get my second dose. | Although I have had my first dose of vaccine, I have not had my second dose. I don’t want to get the next one. | 1: Received, reserved, or intended |
| N/A |  | Although I have had my first dose of vaccine, I have not had my second dose. I want to get the next one. |  |
| N/A | I have had first dose of vaccine, and am waiting for my second dose. | I have had my first dose of vaccine, and am waiting for my second dose. |  |
| N/A | I have had two doses of vaccine. | Although I have had two doses of vaccine, I don’t want to get my booster shot. |  |
| N/A |  | I have got two doses of vaccine. I want to get my booster shot |  |
| N/A | N/A | I have already had two doses of vaccine and a booster shot. |  |

^a^There were three answering options at T1, eight answering options at T2, and thirteen answering options at T3. Multiple answers are not allowed.
