# Supplementary material for: The Impact of Priority Settings at the Start of COVID-19 Mass Vaccination on Subsequent Vaccine Uptake in Japan: One-Year Prospective Cohort Study
Source: JMIR Public Health Surveill. 2023 Jul 10;9:e42143. doi: 10.2196/42143 (PMC10337369; doi:10.2196/42143)
Supplement: Multimedia Appendix 4 [file publichealth_v9i1e42143_app4.docx]

This is a Multimedia Appendix to a full manuscript published in the J Med Internet Res. For full copyright and citation information see http://dx.doi.org/10.2196/42143

**Table S1.** Summary of the presence of pre-existing conditions at T1, February 2021, among the priority group of “18-64 years with pre-existing conditions, non-health care worker,” and their COVID-19 vaccine uptake status at T3 (February 2022; The percentages in parentheses in each category horizontally add up to 100).

|  | Total, n | Received, reserved, or intended, n (%) | Hesitant, n (%) |
| --- | --- | --- | --- |
| **Total** | 1,659 | 1,512 (91.1) | 147 (8.9) |
| **Hypertension^a^** | 779 | 728 (93.5) | 51 (6.5) |
| **Diabetes mellitus^a^** | 326 | 309 (94.8) | 17 (5.2) |
| **Asthma^a^** | 137 | 121 (88.3) | 16 (11.7) |
| **Pneumonia/bronchitis^a^** | 37 | 33 (89.2) | 4 (10.8) |
| **Angina pectoris^a^** | 52 | 45 (86.5) | 7 (13.5) |
| **Myocardial infarction^a^** | 37 | 34 (91.9) | 3 (8.1) |
| **Stroke (including cerebral infarction and cerebral hemorrhage) ^a^** | 49 | 45 (91.8) | 4 (8.2) |
| **Chronic obstructive pulmonary disease^a^** | 29 | 27 (93.1) | 2 (6.9) |
| **Chronic kidney disease^a^** | 51 | 48 (94.1) | 3 (5.9) |
| **Chronic liver disease (excluding fatty liver and hepatitis)^a^** | 35 | 34 (97.1) | 1 (2.9) |
| **Immune disorders and other diseases that cause immune deficiency (including steroid use)^a^** | 79 | 78 (98.7) | 1 (1.3) |
| **Cancer (including malignant tumor)^a^** | 79 | 74 (93.7) | 5 (6.3) |
| **Sleep apnea^b^** | 409 | 268 (91.2) | 141 (8.8) |
| **BMI of ≥30^c^** | 370 | 323 (87.3) | 47 (12.7) |

^a^The pre-exiting conditions were defined if they attended hospital for the disease.

^b^Sleep apnea was defined if they had been identified the disease by medical checkup or physician.

^c^BMI of ≥30 was calculated from the reported height and weight. It is possible that one respondent has multiple pre-existing conditions.

**Table S2**. Summary of job among the priority group of “health care worker”, and their COVID-19 vaccine uptake status (The percentages in parentheses in each category horizontally add up to 100).^a^

|  | Total, n | Received, reserved, or intended, n (%) | Hesitant, n (%) |
| --- | --- | --- | --- |
| **Total** | 831 | 775 (93.3) | 56 (6.7) |
| **Physician** | 55 | 53 (96.4) | 2 (3.6) |
| **Dentist** | 19 | 17 (89.5) | 2 (10.5) |
| **Veterinarian** | 11 | 11 (100) | 0 |
| **Pharmacist** | 63 | 61 (96.8) | 2 (3.2) |
| **Public health nurse** | 5 | 5 (100) | 0 |
| **Midwife** | 7 | 5 (71.4) | 2 (28.6) |
| **Nurse** | 110 | 108 (98.2) | 2 (1.8) |
| **Radiographer** | 13 | 12 (92.3) | 1 (7.7) |
| **Clinical engineer** | 4 | 4 (100) | 0 |
| **Clinical laboratory technician** | 15 | 13 (86.7) | 2 (13.3) |
| **Dental technician** | 8 | 7 (87.5) | 1 (12.5) |
| **Physical therapist** | 32 | 29 (90.6) | 3 (9.4) |
| **Occupational therapist** | 17 | 17 (100) | 0 |
| **Speech therapist** | 6 | 6 (100) | 0 |
| **Dental hygienist** | 11 | 10 (90.9) | 1 (9.1) |
| **Dietitian** | 37 | 36 (97.3) | 1 (2.7) |
| **Practitioner of acupuncture or moxibustion/Judo therapist** | 22 | 18 (81.8) | 4 (18.2) |
| **The other job** | 396 | 363 (91.7) | 33 (8.3) |

^a^Job was derived from T1, February 2021. Vaccine uptake status was derived from T3, February 2022. Multiple answer is not allowed.
