# Supplementary material for: The Impact of Priority Settings at the Start of COVID-19 Mass Vaccination on Subsequent Vaccine Uptake in Japan: One-Year Prospective Cohort Study
Source: JMIR Public Health Surveill. 2023 Jul 10;9:e42143. doi: 10.2196/42143 (PMC10337369; doi:10.2196/42143)
Supplement: Multimedia Appendix 5 [file publichealth_v9i1e42143_app5.docx]

This is a Multimedia Appendix to a full manuscript published in the J Med Internet Res. For full copyright and citation information see http://dx.doi.org/10.2196/42143

**Table S1.** COVID-19 infection over 1 year ago and risk ratios (RRs) for COVID-19 vaccine uptake among total respondents (The percentages in parentheses in each category horizontally add up to 100).^a^

|  |  | Total, n (%) | Received, reserved, or intended, n (%) | Hesitant, n (%) | RR |
| --- | --- | --- | --- | --- | --- |
|  |  |  |  |  |  |
| **Total** |  | 13,555 | 12,237 (90.3) | 1,318 (9.7) |  |
| **COVID-19 infection history over 1 year ago** |  |  |  |  |  |
|  | Never | 13,463 | 12,154 (90.3) | 1,309 (9.7) | 1.00 |
|  | I had | 92 | 83 (90.2) | 9 (9.8) |  |
| **COVID-19 hospitalization over 1 year ago** |  |  |  |  |  |
|  | Never | 13,488 | 12,178 (90.3) | 1,310 (9.7) | 0.98 |
|  | I had | 67 | 59 (88.1) | 8 (11.9) |  |

^a^Vaccine uptake status, COVID-19 infection and COVID-19 hospitalization over 1 year ago were derived from T3, February 2022.

**Table S2.** COVID-19 infection over 1 year ago and risk ratios (RRs) for COVID-19 vaccine uptake among the nonpriority group (The percentages in parentheses in each category horizontally add up to 100).^a^

|  |  | Total, n (%) | Received, reserved, or intended, n (%) | Hesitant, n (%) | RR |
| --- | --- | --- | --- | --- | --- |
|  |  |  |  |  |  |
| **Total** |  | 7,017 | 6,085 (86.7) | 932 (13.3) |  |
| **COVID-19 infection history over 1 year ago** |  |  |  |  |  |
|  | Never | 6,964 | 6,039 (86.7) | 925 (13.3) | 1.00 |
|  | I had | 53 | 46 (86.8) | 7 (13.2) |  |
| **COVID-19 hospitalization over 1 year ago** |  |  |  |  |  |
|  | Never | 6,984 | 6,057 (86.7) | 927 (13.3) | 0.98 |
|  | I had | 33 | 28 (84.8) | 5 (15.2) |  |

^a^Priority setting was derived from T1, February 2021. Vaccine uptake status, COVID-19 infection and COVID-19 hospitalization over 1 year ago were derived from T3, February 2022.

**Table S3.** COVID-19 infection over 1 year ago and risk ratios (RRs) for COVID-19 vaccine uptake among the priority group of 18-64 years with pre-existing conditions, non-health care worker (The percentages in parentheses in each category horizontally add up to 100).^a^

|  |  | Total, n (%) | Received, reserved, or intended, n (%) | Hesitant, n (%) | RR |
| --- | --- | --- | --- | --- | --- |
|  |  |  |  |  |  |
| **Total** |  | 1,659 | 1,512 (91.1) | 147 (8.9) |  |
| **COVID-19 infection history over 1 year ago** |  |  |  |  |  |
|  | Never | 1,644 | 1,497 (91.1) | 147 (8.9) | 1.10 |
|  | I had | 15 | 15 (100) | 0 |  |
| **COVID-19 hospitalization over 1 year ago** |  |  |  |  |  |
|  | Never | 1,644 | 1,498 (91.1) | 146 (8.9) | 1.02 |
|  | I had | 15 | 14 (93.3) | 1 (6.7) |  |

^a^Priority setting was derived from T1, February 2021. Vaccine uptake status, COVID-19 infection and COVID-19 hospitalization over 1 year ago were derived from T3, February 2022.

**Table S4.** COVID-19 infection over 1 year ago and risk ratios (RRs) for COVID-19 vaccine uptake among the priority group of ≥65 years, non-health care worker (The percentages in parentheses in each category horizontally add up to 100).^a^

|  |  | Total, n (%) | Received, reserved, or intended, n (%) | Hesitant, n (%) | RR |
| --- | --- | --- | --- | --- | --- |
|  |  |  |  |  |  |
| **Total** |  | 4,048 | 3,865 (95.5) | 183 (4.5) |  |
| **COVID-19 infection history over 1 year ago** |  |  |  |  |  |
|  | Never | 4,041 | 3,859 (95.5) | 182 (4.5) | 0.90 |
|  | I had | 7 | 6 (85.7) | 1 (14.3) |  |
| **COVID-19 hospitalization over 1 year ago** |  |  |  |  |  |
|  | Never | 4,043 | 3,860 (95.5) | 183 (4.5) | 1.05 |
|  | I had | 5 | 5 (100) | 0 |  |

^a^Priority setting was derived from T1, February 2021. Vaccine uptake status, COVID-19 infection and COVID-19 hospitalization over 1 year ago were derived from T3, February 2022.

**Table S5.** COVID-19 infection over 1 year ago and risk ratios (RRs) for COVID-19 vaccine uptake among the priority group of health care worker (The percentages in parentheses in each category horizontally add up to 100).^a^

|  |  | Total, n (%) | Received, reserved, or intended, n (%) | Hesitant, n (%) | RR |
| --- | --- | --- | --- | --- | --- |
|  |  |  |  |  |  |
| **Total** |  | 831 | 775 (93.3) | 56 (6.7) |  |
| **COVID-19 infection history over 1 year ago** |  |  |  |  |  |
|  | Never | 814 | 759 (93.2) | 55 (6.8) | 1.01 |
|  | I had | 17 | 16 (94.1) | 1 (5.9) |  |
| **COVID-19 hospitalization over 1 year ago** |  |  |  |  |  |
|  | Never | 817 | 763 (93.4) | 54 (6.6) | 0.92 |
|  | I had | 14 | 12 (85.7) | 2 (14.3) |  |

^a^Priority setting was derived from T1, February 2021. Vaccine uptake status, COVID-19 infection and COVID-19 hospitalization over 1 year ago were derived from T3, February 2022.
