# Supplementary material for: The Impact of Priority Settings at the Start of COVID-19 Mass Vaccination on Subsequent Vaccine Uptake in Japan: One-Year Prospective Cohort Study
Source: JMIR Public Health Surveill. 2023 Jul 10;9:e42143. doi: 10.2196/42143 (PMC10337369; doi:10.2196/42143)
Supplement: Multimedia Appendix 6 [file publichealth_v9i1e42143_app6.pptx]

## Slide 1
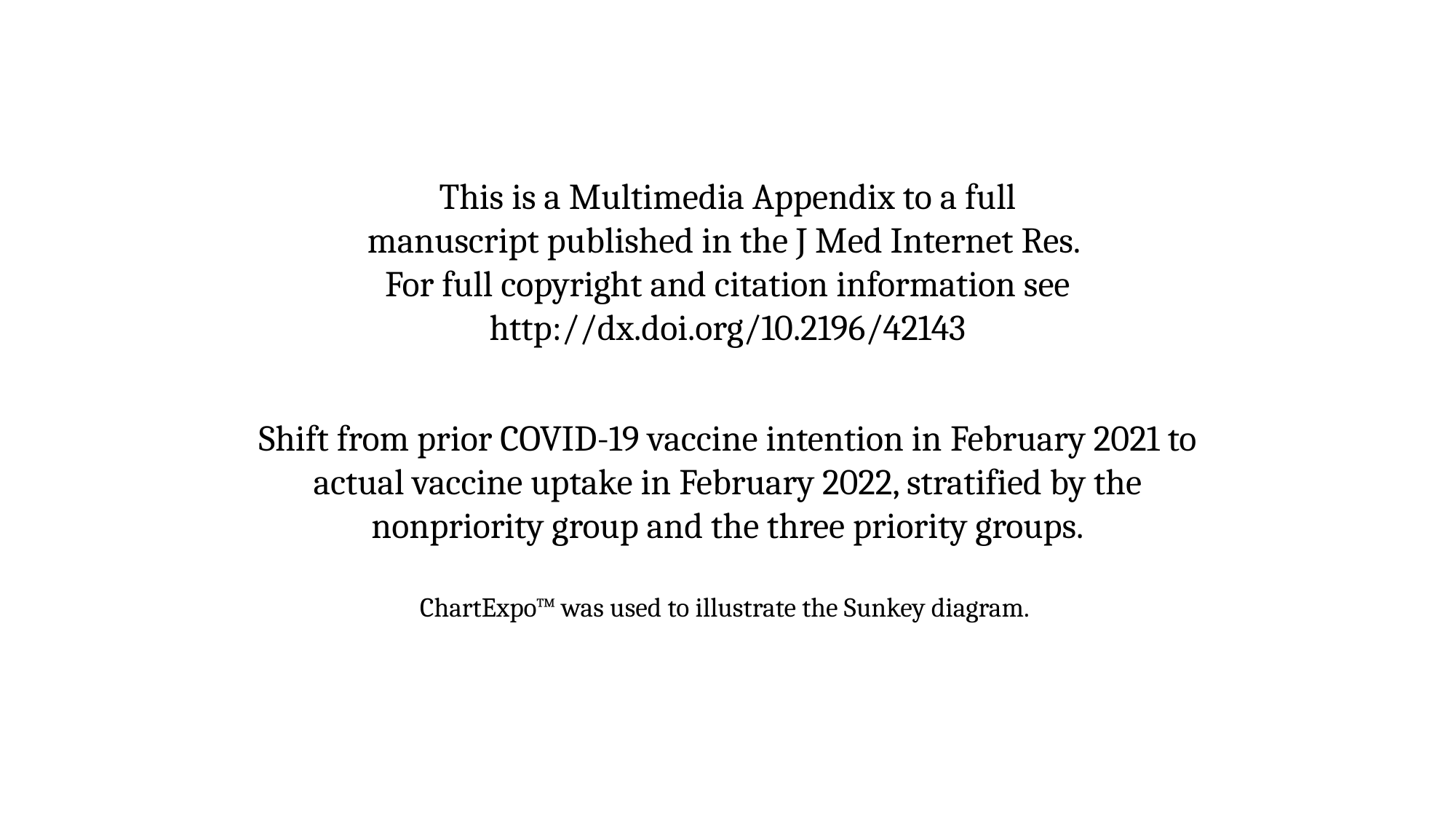

This is a Multimedia Appendix to a full manuscript published in the J Med Internet Res.
For full copyright and citation information see http://dx.doi.org/10.2196/42143
Shift from prior COVID-19 vaccine intention in February 2021 to actual vaccine uptake in February 2022, stratified by the nonpriority group and the three priority groups.
ChartExpo™ was used to illustrate the Sunkey diagram.

## Slide 2
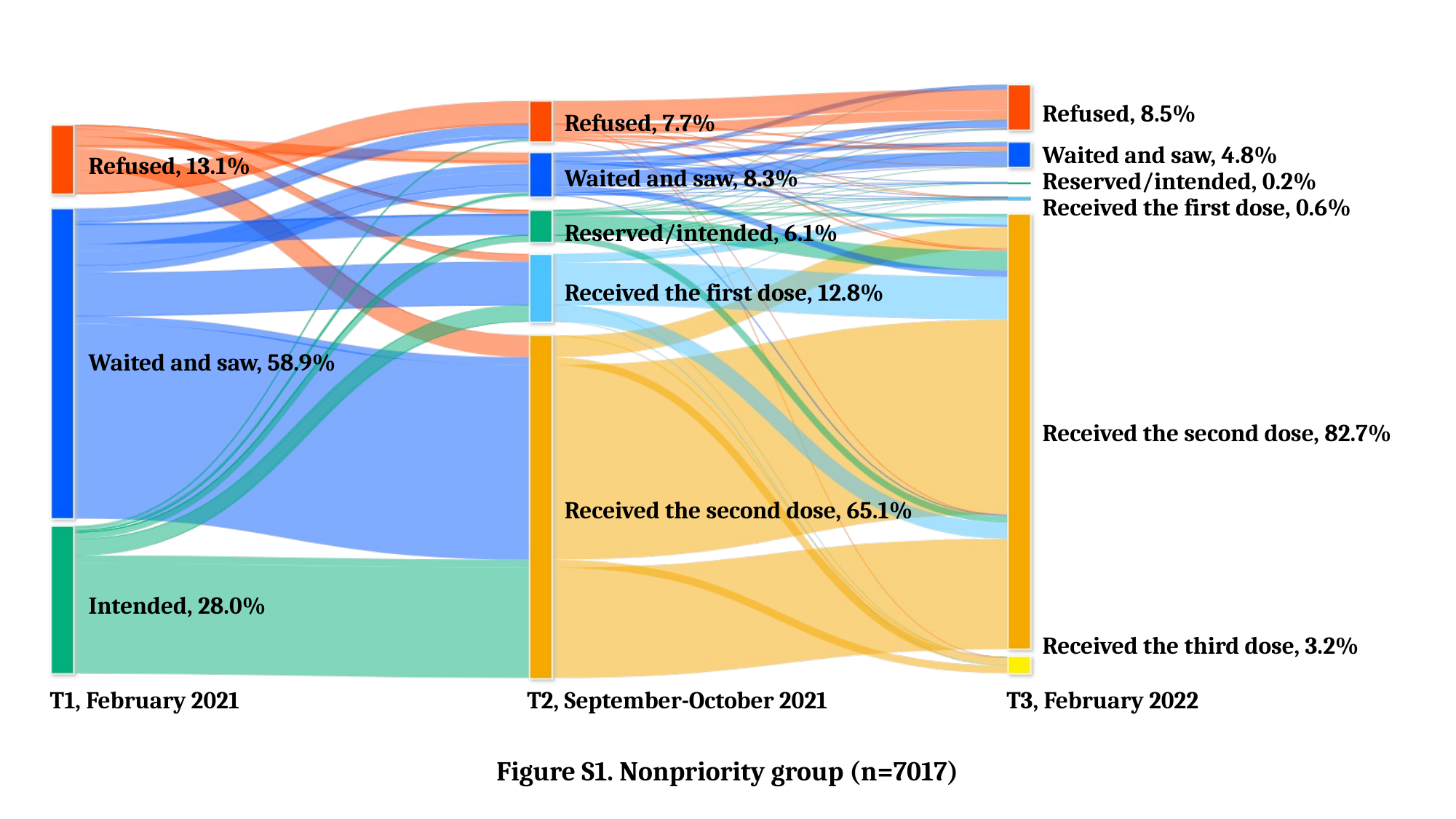

Refused, 8.5%
Refused, 7.7%
Waited and saw, 4.8%
Refused, 13.1%
Waited and saw, 8.3%
Reserved/intended, 0.2%
Received the first dose, 0.6%
Reserved/intended, 6.1%
Received the first dose, 12.8%
Waited and saw, 58.9%
Received the second dose, 82.7%
Received the second dose, 65.1%
Intended, 28.0%
Received the third dose, 3.2%
T1, February 2021
T2, September-October 2021
T3, February 2022
Figure S1. Nonpriority group (n=7017)

## Slide 3
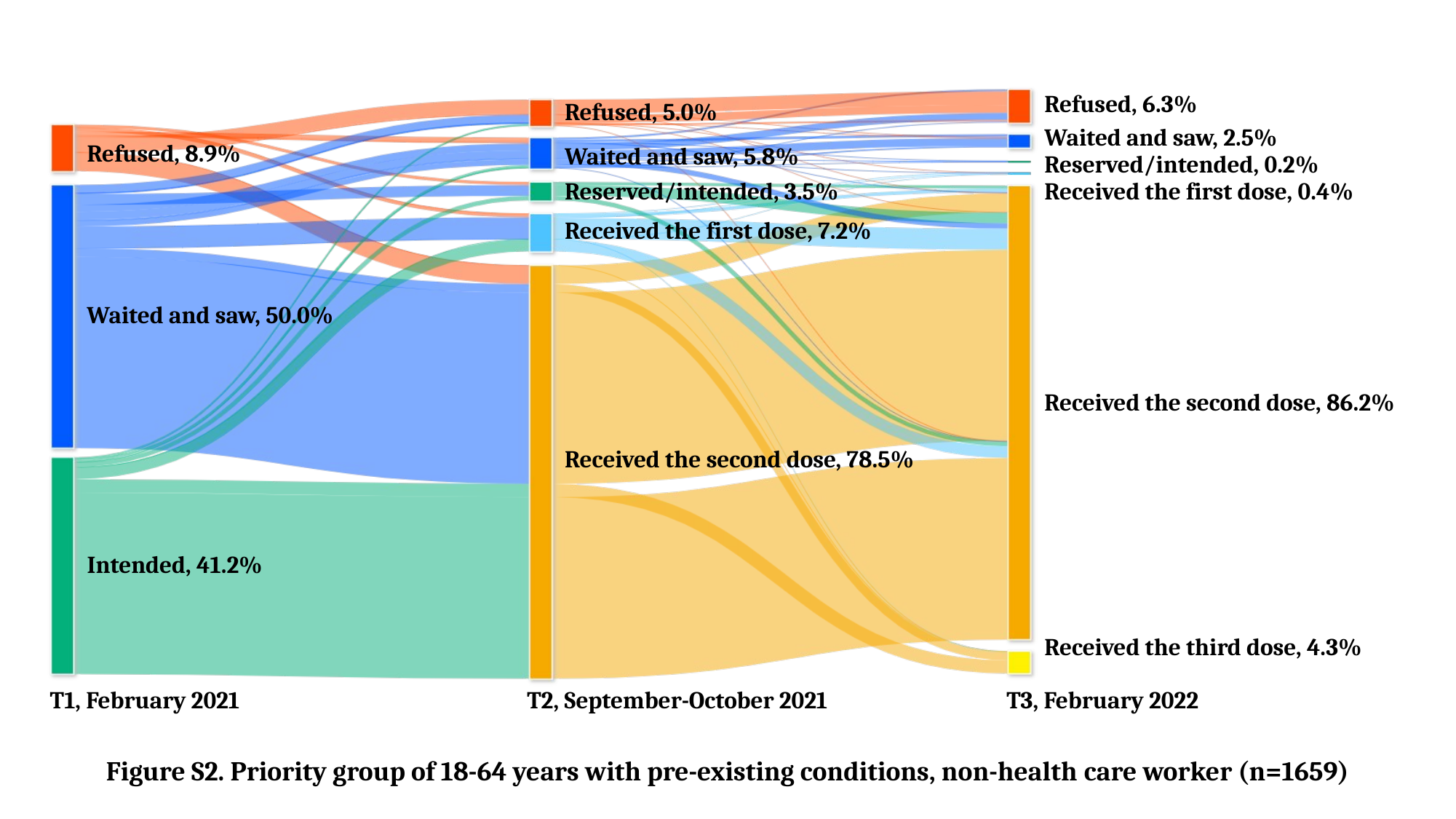

Refused, 6.3%
Refused, 5.0%
Waited and saw, 2.5%
Refused, 8.9%
Waited and saw, 5.8%
Reserved/intended, 0.2%
Reserved/intended, 3.5%
Received the first dose, 0.4%
Received the first dose, 7.2%
Waited and saw, 50.0%
Received the second dose, 86.2%
Received the second dose, 78.5%
Intended, 41.2%
Received the third dose, 4.3%
T1, February 2021
T2, September-October 2021
T3, February 2022
Figure S2. Priority group of 18-64 years with pre-existing conditions, non-health care worker (n=1659)

## Slide 4
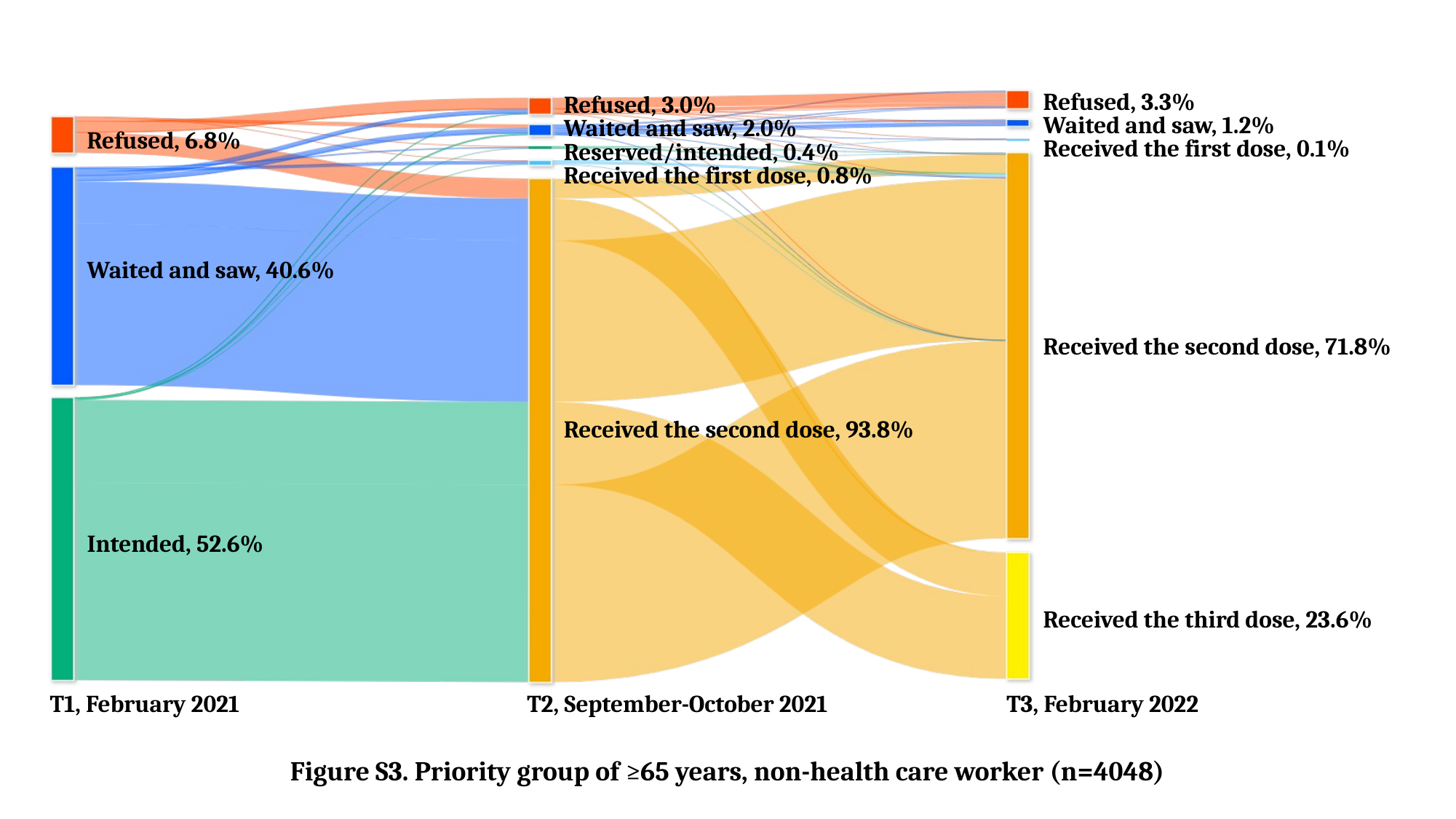

Refused, 3.3%
Refused, 3.0%
Waited and saw, 1.2%
Waited and saw, 2.0%
Refused, 6.8%
Received the first dose, 0.1%
Reserved/intended, 0.4%
Received the first dose, 0.8%
Waited and saw, 40.6%
Received the second dose, 71.8%
Received the second dose, 93.8%
Intended, 52.6%
Received the third dose, 23.6%
T1, February 2021
T2, September-October 2021
T3, February 2022
Figure S3. Priority group of ≥65 years, non-health care worker (n=4048)

## Slide 5
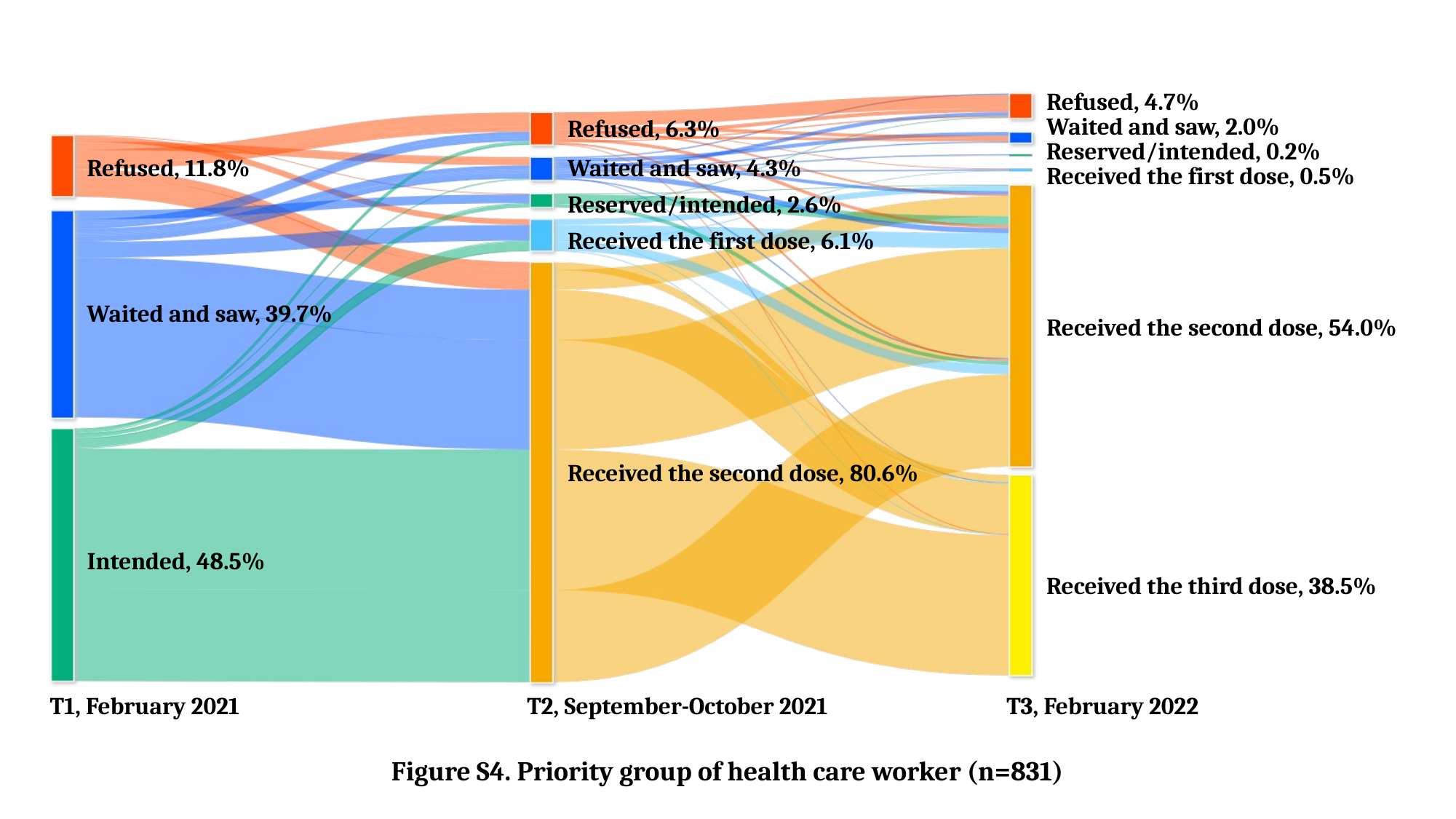

Refused, 4.7%
Waited and saw, 2.0%
Refused, 6.3%
Reserved/intended, 0.2%
Waited and saw, 4.3%
Refused, 11.8%
Received the first dose, 0.5%
Reserved/intended, 2.6%
Received the first dose, 6.1%
Waited and saw, 39.7%
Received the second dose, 54.0%
Received the second dose, 80.6%
Intended, 48.5%
Received the third dose, 38.5%
T1, February 2021
T2, September-October 2021
T3, February 2022
Figure S4. Priority group of health care worker (n=831)
