# Supplementary material for: The Impact of Priority Settings at the Start of COVID-19 Mass Vaccination on Subsequent Vaccine Uptake in Japan: One-Year Prospective Cohort Study
Source: JMIR Public Health Surveill. 2023 Jul 10;9:e42143. doi: 10.2196/42143 (PMC10337369; doi:10.2196/42143)
Supplement: Multimedia Appendix 7 [file publichealth_v9i1e42143_app7.pptx]

## Slide 1
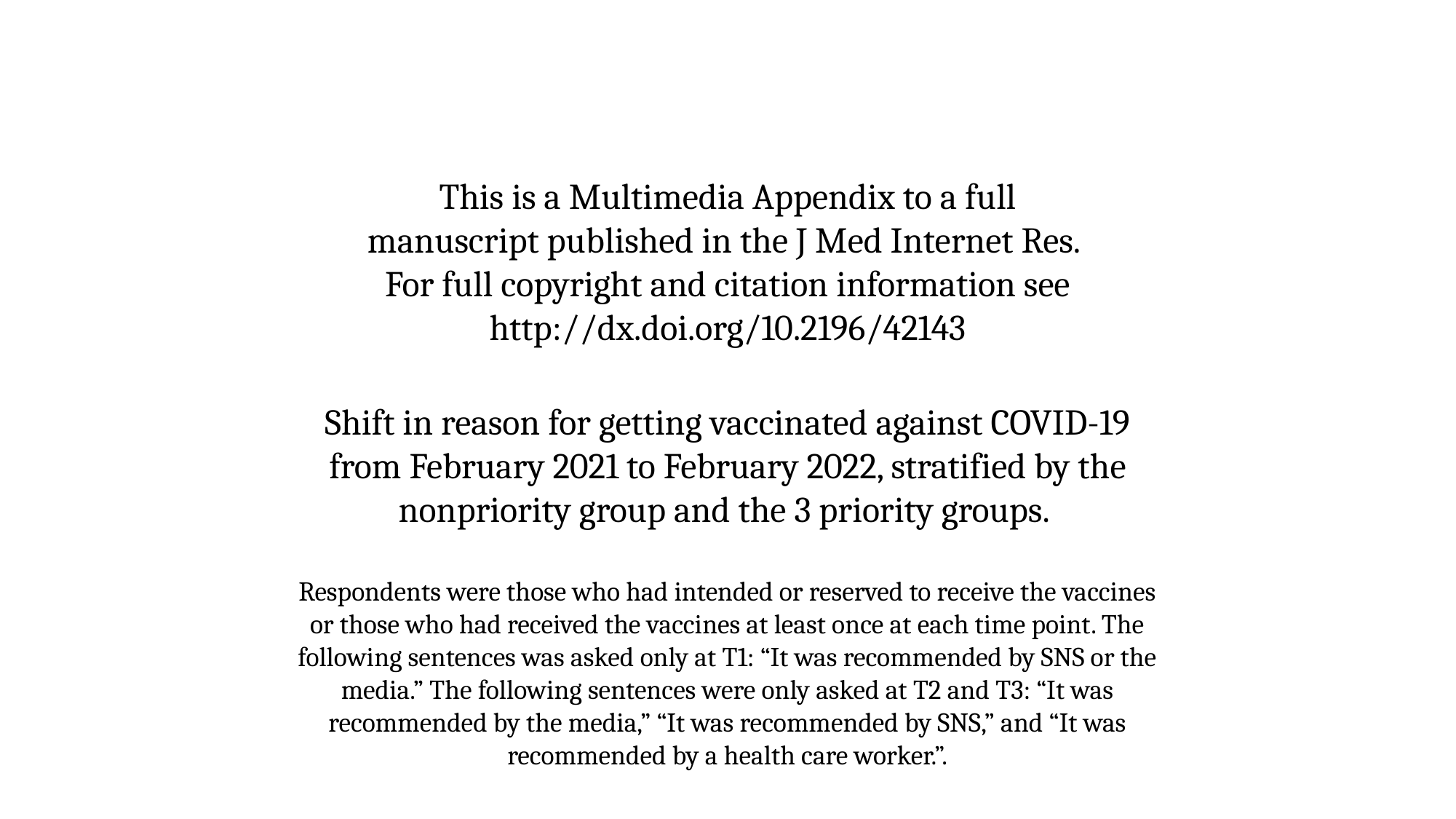

This is a Multimedia Appendix to a full manuscript published in the J Med Internet Res.
For full copyright and citation information see http://dx.doi.org/10.2196/42143
Shift in reason for getting vaccinated against COVID-19 from February 2021 to February 2022, stratified by the nonpriority group and the 3 priority groups.
Respondents were those who had intended or reserved to receive the vaccines or those who had received the vaccines at least once at each time point. The following sentences was asked only at T1: “It was recommended by SNS or the media.” The following sentences were only asked at T2 and T3: “It was recommended by the media,” “It was recommended by SNS,” and “It was recommended by a health care worker.”.

## Slide 2
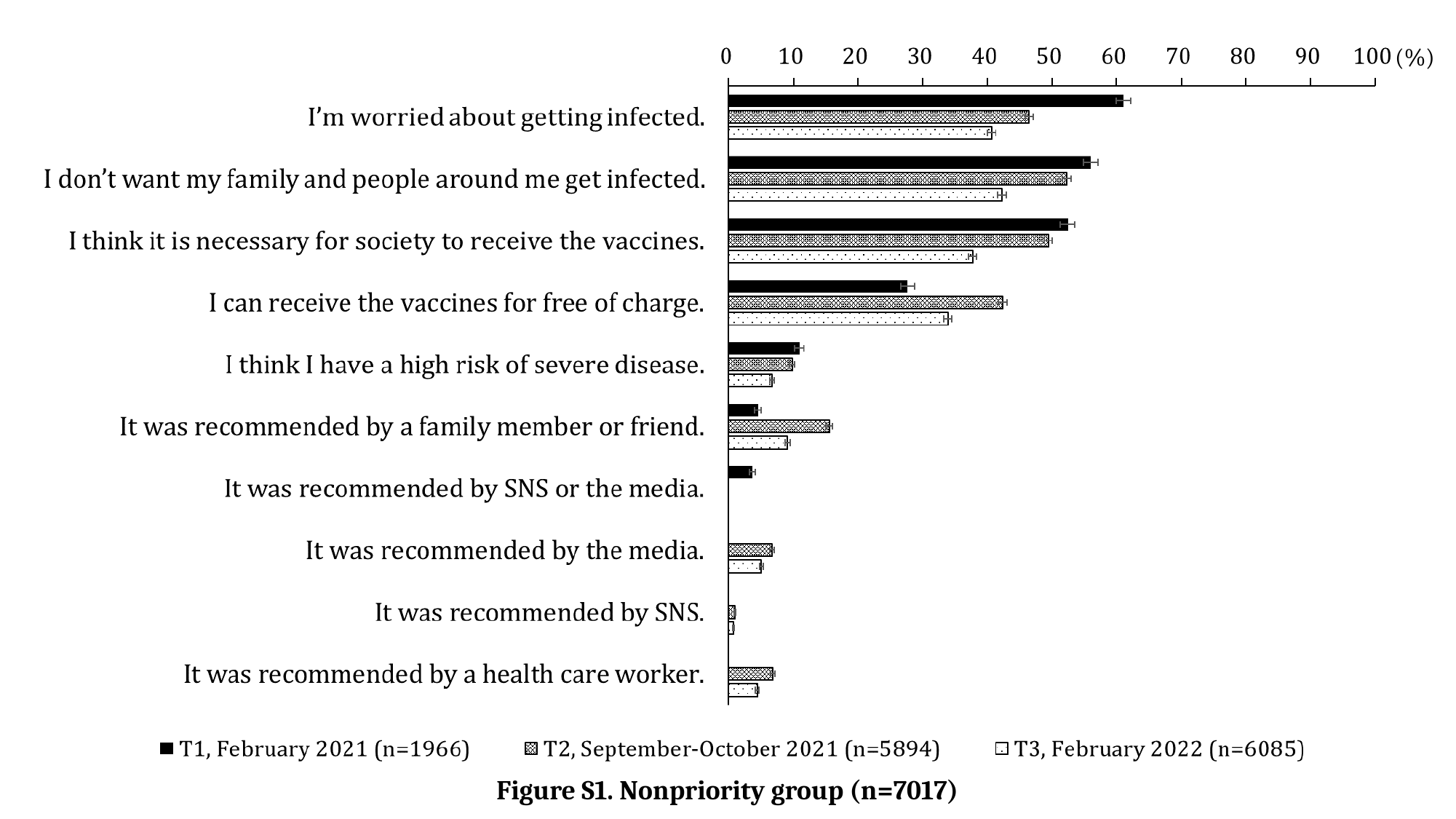

(%)
Figure S1. Nonpriority group (n=7017)

## Slide 3
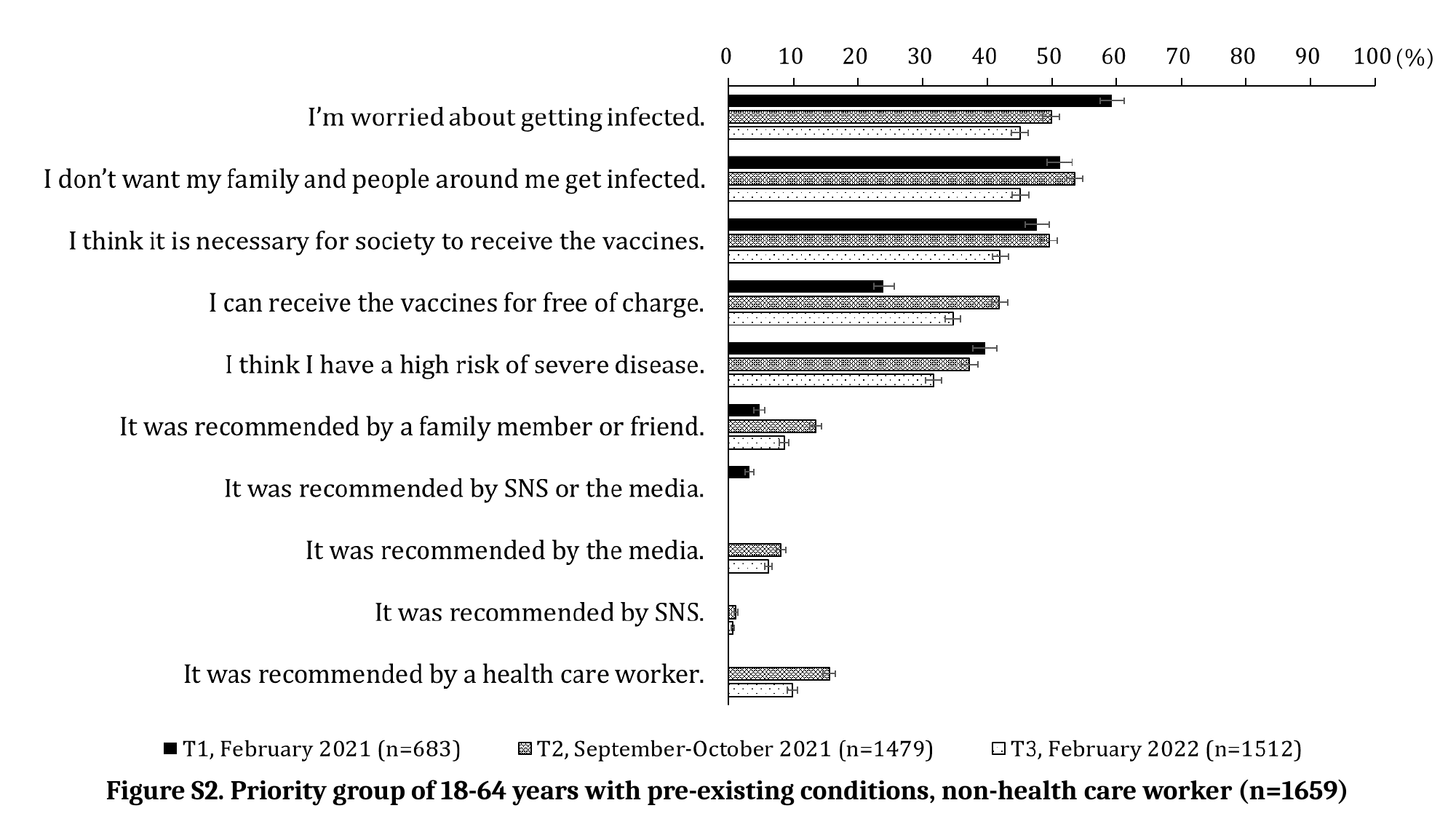

(%)
Figure S2. Priority group of 18-64 years with pre-existing conditions, non-health care worker (n=1659)

## Slide 4
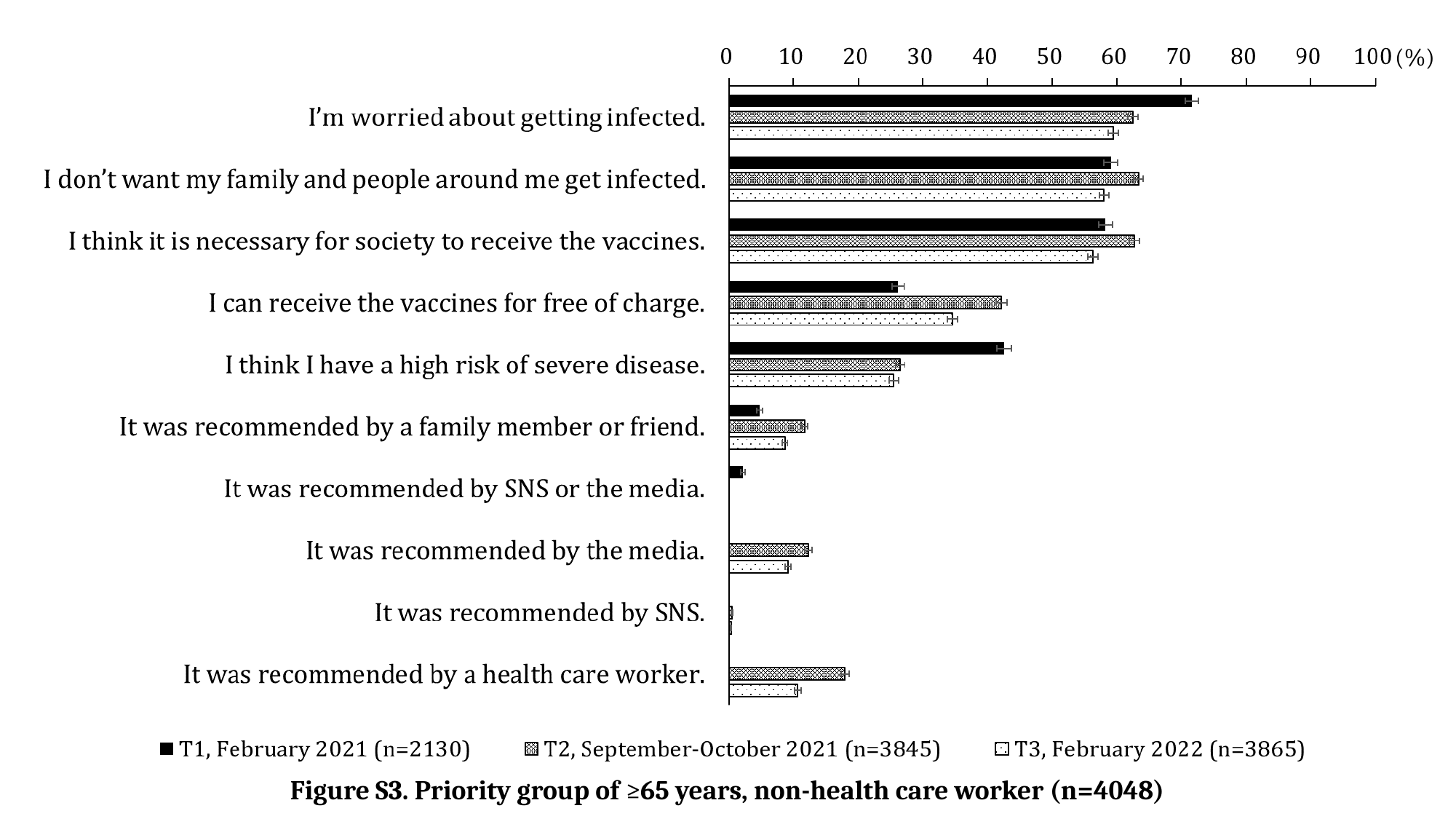

(%)
Figure S3. Priority group of ≥65 years, non-health care worker (n=4048)

## Slide 5
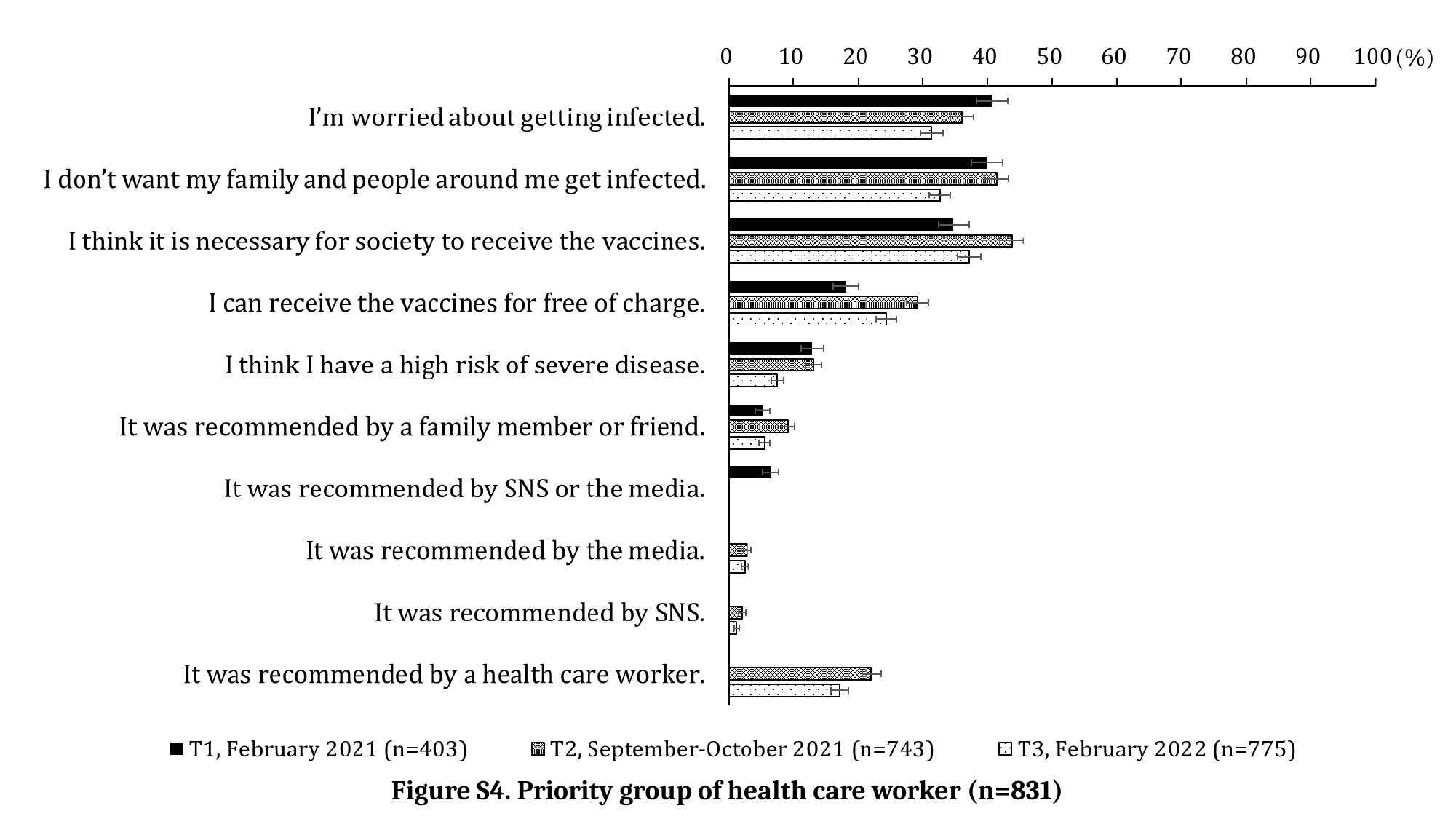

(%)
Figure S4. Priority group of health care worker (n=831)
