# Supplementary material for: The Impact of Priority Settings at the Start of COVID-19 Mass Vaccination on Subsequent Vaccine Uptake in Japan: One-Year Prospective Cohort Study
Source: JMIR Public Health Surveill. 2023 Jul 10;9:e42143. doi: 10.2196/42143 (PMC10337369; doi:10.2196/42143)
Supplement: Multimedia Appendix 8 [file publichealth_v9i1e42143_app8.pptx]

## Slide 1
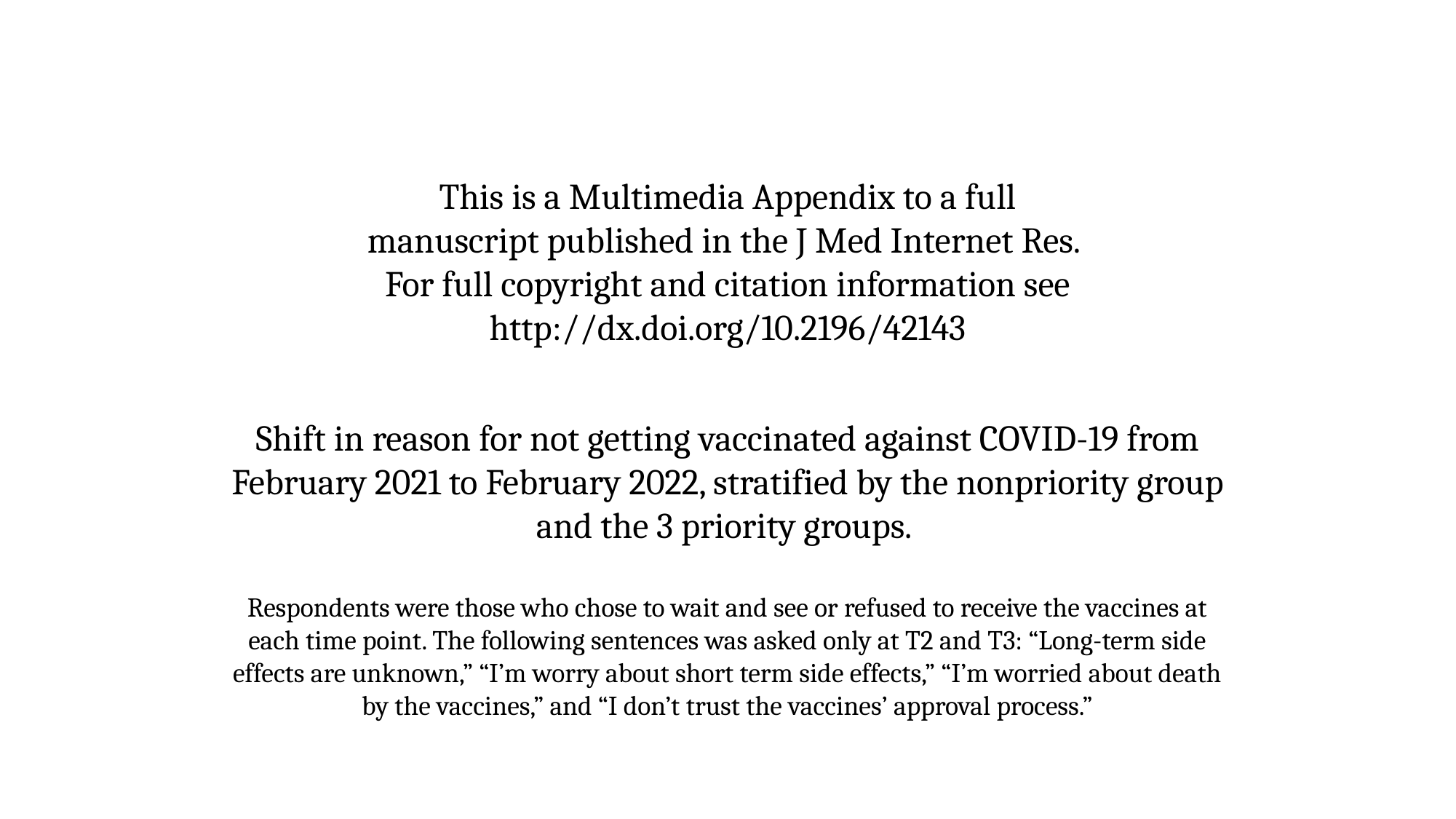

This is a Multimedia Appendix to a full manuscript published in the J Med Internet Res.
For full copyright and citation information see http://dx.doi.org/10.2196/42143
Shift in reason for not getting vaccinated against COVID-19 from February 2021 to February 2022, stratified by the nonpriority group and the 3 priority groups.
Respondents were those who chose to wait and see or refused to receive the vaccines at each time point. The following sentences was asked only at T2 and T3: “Long-term side effects are unknown,” “I’m worry about short term side effects,” “I’m worried about death by the vaccines,” and “I don’t trust the vaccines’ approval process.”

## Slide 2
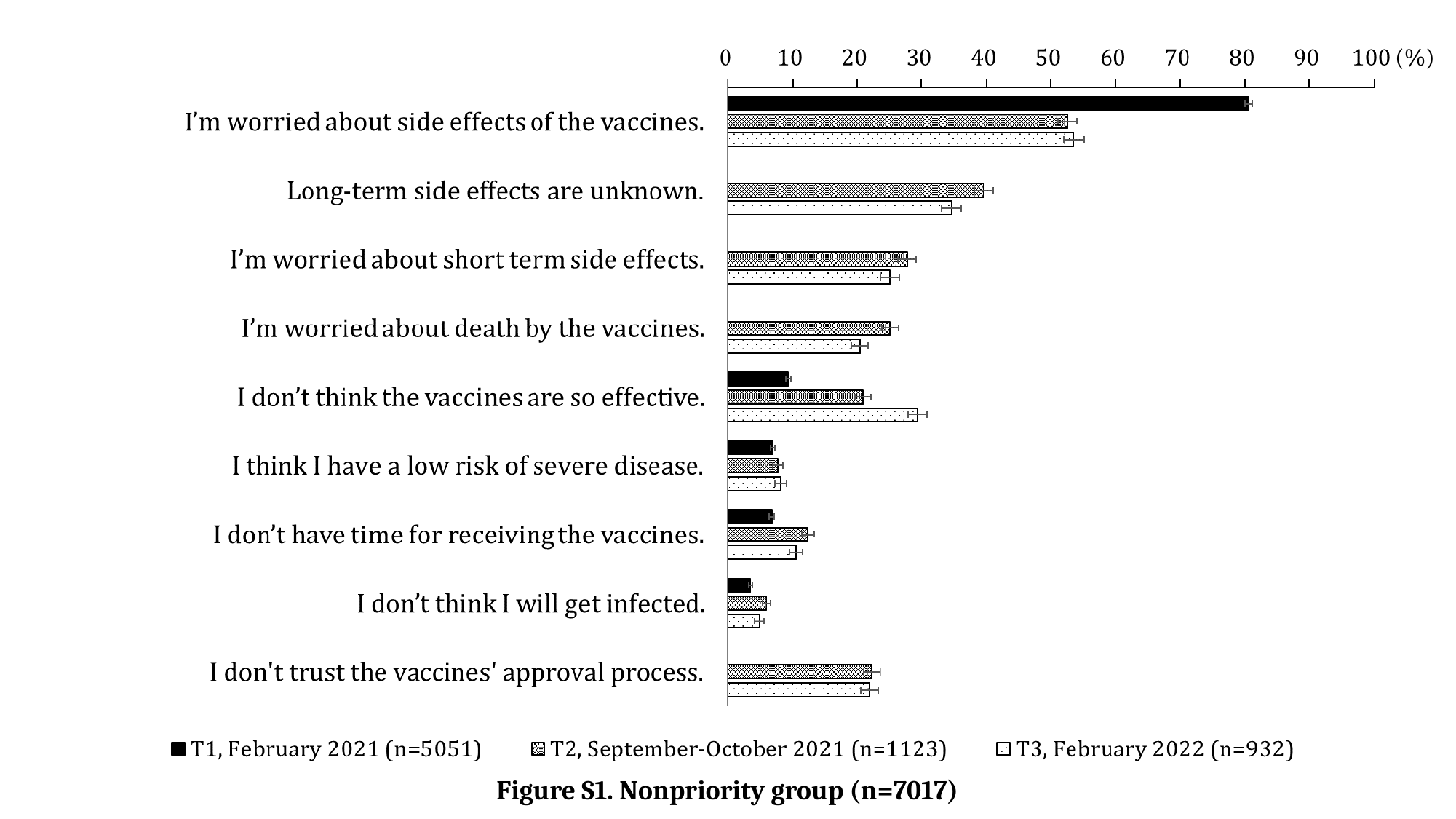

(%)
Figure S1. Nonpriority group (n=7017)

## Slide 3
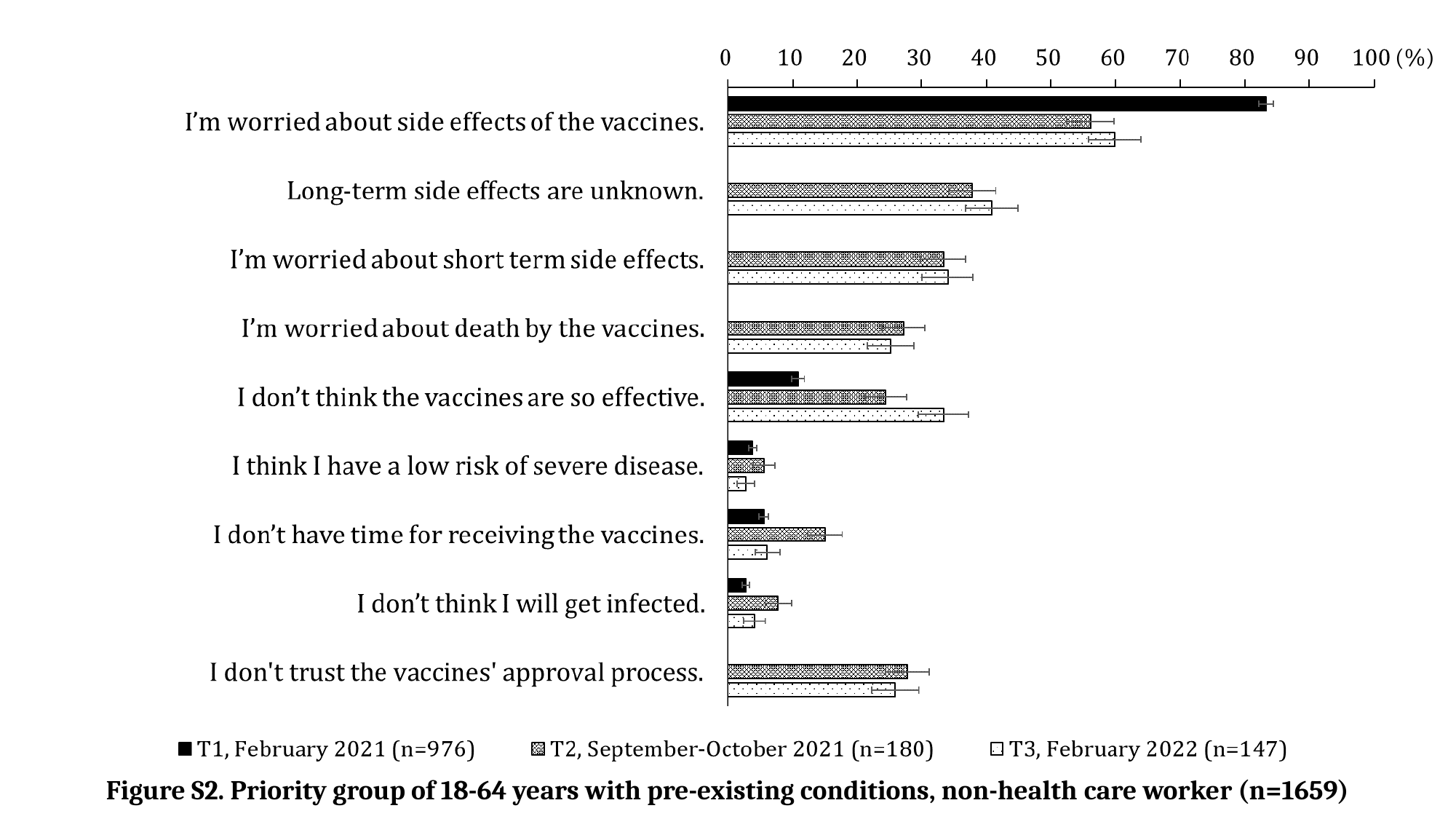

(%)
Figure S2. Priority group of 18-64 years with pre-existing conditions, non-health care worker (n=1659)

## Slide 4
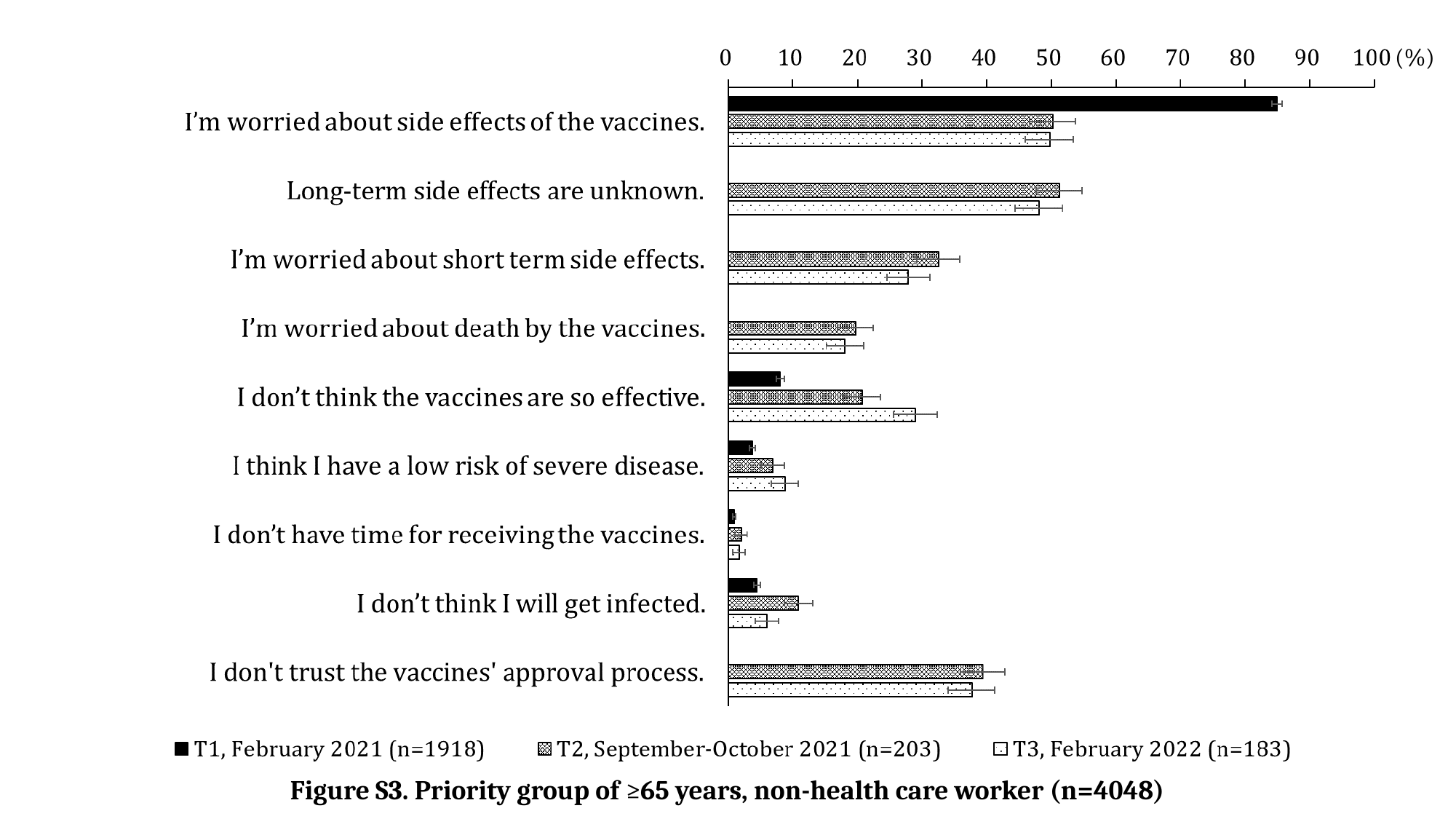

(%)
Figure S3. Priority group of ≥65 years, non-health care worker (n=4048)

## Slide 5
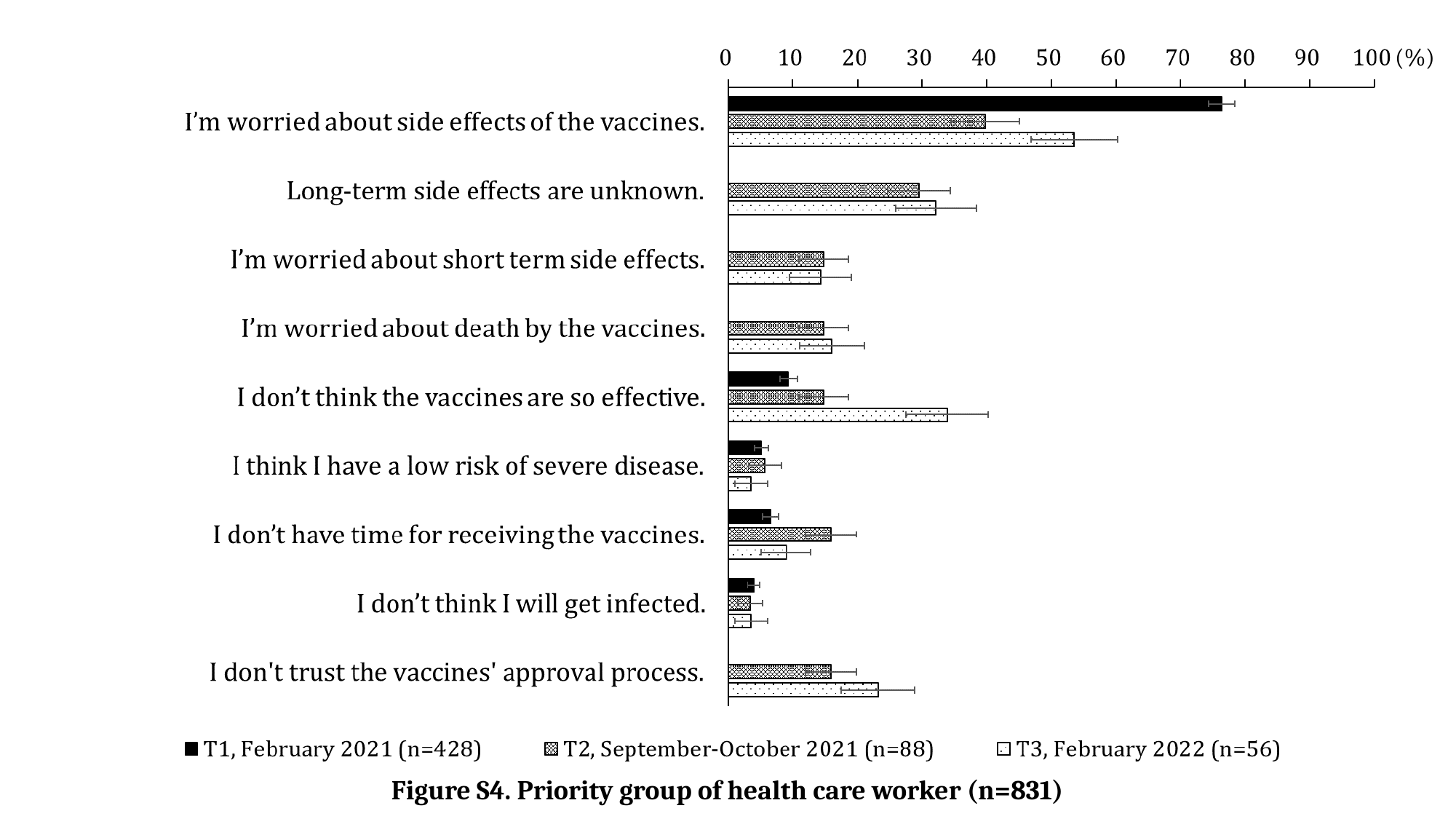

(%)
Figure S4. Priority group of health care worker (n=831)
