# Supplementary material for: The Impact of Priority Settings at the Start of COVID-19 Mass Vaccination on Subsequent Vaccine Uptake in Japan: One-Year Prospective Cohort Study
Source: JMIR Public Health Surveill. 2023 Jul 10;9:e42143. doi: 10.2196/42143 (PMC10337369; doi:10.2196/42143)
Supplement: Multimedia Appendix 9 [file publichealth_v9i1e42143_app9.docx]

This is a Multimedia Appendix to a full manuscript published in the J Med Internet Res. For full copyright and citation information see http://dx.doi.org/10.2196/42143

**Table S1.** Risk ratios (RRs) for COVID-19 vaccination uptake status at T3 (February 2022) using sociodemographic, behavioral factors, and vaccine hesitancy-related scales.^a^

| Explanatory variables | | Total | Received, reserved, or intended^b^,  n (%) | Model 1, RR (95% CI) | Model 2, RR (95% CI) | Model 3, RR (95% CI) | Model 4, RR (95% CI) |
| --- | --- | --- | --- | --- | --- | --- | --- |
|  |  |  |  |  |  |  |  |
| **Prior COVID-19 vaccination intention**^c^ | |  |  |  |  |  |  |
|  | Intended | 5,182 | 5,105 (98.5) | - | 1.67 (1.60-1.75) | 1.45 (1.39-1.52) | 1.45 (1.39-1.52) |
|  | Waited and saw | 6,936 | 6,320 (91.1) | - | 1.58 (1.51-1.65) | 1.43 (1.37-1.49) | 1.43 (1.37-1.49) |
|  | Refused | 1,437 | 812 (56.5) | - | Reference | Reference | Reference |
| **Age group**^c^ | |  |  |  |  |  |  |
|  | 18-34 | 2,035 | 1,717 (84.4) | Reference | Reference | Reference | Reference |
|  | 35-44 | 2,052 | 1,750 (85.3) | 1.01 (0.98-1.03) | 0.99 (0.97-1.02) | 0.99 (0.97-1.02) | 0.99 (0.97-1.02) |
|  | 45-54 | 2,774 | 2,458 (88.6) | 1.04 (1.02-1.07) | 1.04 (1.01-1.06) | 1.01 (0.99-1.04) | 1.02 (0.99-1.04) |
|  | 55-64 | 2,600 | 2,403 (92.4) | 1.09 (1.07-1.11) | 1.05 (1.03-1.07) | 1.01 (0.99-1.03) | 1.01 (0.99-1.03) |
|  | 65-80 | 4,094 | 3,909 (95.5) | 1.15 (1.12-1.17) | 1.09 (1.06-1.11) | 1.03 (1.01-1.05) | 1.03 (1.01-1.05) |
| **Employment status**^c^ | |  |  |  |  |  |  |
|  | Employed, non-HCW^d^ | 7,209 | 6,480 (89.9) | Reference | Reference | Reference | Reference |
|  | Employed, HCW | 831 | 775 (93.3) | 1.06 (1.04-1.08) | 1.03 (1.01-1.05) | 1.04 (1.02-1.06) | 1.04 (1.02-1.06) |
|  | Unemployed | 2,034 | 1,793 (88.2) | 0.92 (0.90-0.94) | 0.96 (0.94-0.97) | 0.96 (0.94-0.97) | 0.96 (0.94-0.97) |
|  | Not working (student/homemaker/retire) | 3,481 | 3,189 (91.6) | 0.98 (0.97-0.996) | 0.98 (0.97-0.995) | 0.99 (0.97-0.999) | 0.99 (0.97-0.999) |
| **Number of pre-existing conditions**^c^ | |  |  |  |  |  |  |
|  | 0 | 9,860 | 8,740 (88.6) | Reference | Reference | Reference | Reference |
|  | 1+ | 3,695 | 3,497 (94.6) | 1.04 (1.03-1.05) | 1.02 (1.01-1.03) | 1.01 (1.003-1.02) | 1.01 (1.003-1.02) |
| **Sex**^c^ |  |  |  |  |  |  |  |
|  | Male | 7,036 | 6,369 (90.5) | - | Reference | Reference | Reference |
|  | Female | 6,519 | 5,868 (90.0) | - | 1.03 (1.01-1.04) | 1.02 (1.01-1.03) | 1.02 (1.01-1.03) |
| **Marital status**^c^ | |  |  |  |  |  |  |
|  | Single/divorced/widowed | 4,945 | 4,231 (85.6) | - | Reference | Reference | Reference |
|  | Married | 8,610 | 8,006 (93.0) | - | 1.03 (1.01-1.04) | 1.02 (1.01-1.03) | 1.02 (1.01-1.03) |
| **Educational background**^c^ | |  |  |  |  |  |  |
|  | Others | 6,811 | 6,066 (89.1) | - | Reference | Reference | Reference |
|  | 4-year college/university/graduate | 6,744 | 6,171 (91.5) | - | 1.03 (1.01-1.04) | 1.02 (1.01-1.03) | 1.02 (1.01-1.03) |
| **Household income (million yen)**^c^ | |  |  |  |  |  |  |
|  | <5 | 5,530 | 4,923 (89.0) | - | Reference | Reference | Reference |
|  | 5 to 10 | 4,048 | 3,724 (92.0) | - | 1.02 (1.01-1.04) | 1.02 (1.003-1.03) | 1.02 (1.003-1.03) |
|  | ≥10 | 1,383 | 1,298 (93.9) | - | 1.03 (1.01-1.05) | 1.02 (1.01-1.04) | 1.02 (1.01-1.04) |
|  | I do not know/Prefer not to answer. | 2,594 | 2,292 (88.4) | - | 1.01 (0.995-1.03) | 1.01 (0.997-1.03) | 1.01 (0.997-1.03) |
| **Influenza vaccination in 2019/2020 season**^c^ | |  |  |  |  |  |  |
|  | No | 8,246 | 7,107 (86.2) | - | Reference | Reference | Reference |
|  | Yes | 5,309 | 5,130 (96.6) | - | 1.04 (1.04-1.05) | 1.02 (1.02-1.03) | 1.02 (1.02-1.03) |
| **Regular medical checkups before pandemic**^e^ | |  |  |  |  |  |  |
|  | No | 4,612 | 3,856 (83.6) | - | - | Reference | Reference |
|  | Yes | 8,943 | 8,381 (93.7) | - | - | 1.04 (1.03-1.05) | 1.04 (1.03-1.05) |
| **Aversion to risk of vaccine**^e^ | |  |  |  |  |  |  |
|  | Low, 1.00-3.00 | 6,587 | 6,146 (93.3) | - | - | 1.05 (1.04-1.06) | 1.05 (1.04-1.06) |
|  | High, 3.01-5.00 | 6,968 | 6,091 (87.4) | - | - | Reference | Reference |
| **Lack of confidence in vaccine**^e^ | |  |  |  |  |  |  |
|  | Low, 1.00-1.99 | 4,080 | 4,049 (99.2) | - | - | 1.22 (1.20-1.24) | 1.22 (1.20-1.24) |
|  | Middle, 2.00-2.43 | 5,458 | 5,290 (96.9) | - | - | 1.23 (1.21-1.25) | 1.23 (1.21-1.25) |
|  | High, 2.44-5.00 | 4,017 | 2,898 (72.1) | - | - | Reference | Reference |
| **Conspiracy beliefs in vaccine**^e^ | |  |  |  |  |  |  |
|  | Low, 1.00-3.27 | 4,253 | 4,142 (97.4) | - | - | 1.08 (1.06-1.10) | 1.08 (1.06-1.10) |
|  | Middle, 3.28-4.00 | 6,399 | 5,865 (91.7) | - | - | 1.09 (1.07-1.11) | 1.09 (1.07-1.11) |
|  | High, 4.01-5.00 | 2,903 | 2,230 (76.8) | - | - | Reference | Reference |
| **Fear of COVID-19**^e^ | |  |  |  |  |  |  |
|  | Low, 1.00-2.27 | 3,756 | 3,233 (86.1) | - | - | 0.96 (0.94-0.97) | 0.96 (0.94-0.97) |
|  | Middle, 2.28-3.00 | 6,638 | 6,086 (91.7) | - | - | 0.99 (0.98-1.01) | 0.99 (0.98-1.01) |
|  | High, 3.01-7.00 | 3,161 | 2,918 (92.3) | - | - | Reference | Reference |
| **COVID-19 infection history**^b^ | |  |  |  |  |  |  |
|  | Never | 13,264 | 11,978 (90.3) | - | - |  | Reference |
|  | I had | 291 | 259 (89.0) | - | - |  | 1.03 (0.99-1.08) |
| **Akaike's Information Criterion** | |  |  | 26953.524 | 26736.114 | 26636.506 | 26638.231 |

^a^Modified Poisson regression analysis with a robust error variance was performed. Higher RR showed higher likelihood of received, reserved, or intended vaccination status. In Model 1, age group, employment status, and number of pre-existing conditions were entered. In addition to Model 1, Model 2 was adjusted for prior vaccination intention, sex, marital status, educational background, annual household income, influenza vaccination in 2019/2020 season. In addition to Model 2, Model 3 was adjusted for regular medical checkups before pandemic, aversion to risk of vaccine, lack of confidence in vaccine, conspiracy beliefs in vaccine, fear of COVID-19. In addition to Model 3, Model 4 was adjusted for COVID-19 infection history.

^b^The variables were derived from T3, February 2022.

^c^The variables were derived from T1, February 2021.

^d^HCW healthcare worker.

^e^The variables were derived from T2, September-October 2021.
